# Structural and mechanistic profiling of Nurr1 modulation by vidofludimus enables structure-guided ligand design

**DOI:** 10.1038/s42004-025-01553-8

**Published:** 2025-05-21

**Authors:** Úrsula López-García, Jan Vietor, Julian A. Marschner, Jan Heering, Vasily Morozov, Thomas Wein, Daniel Merk

**Affiliations:** 1 Department of Pharmacy, Ludwig-Maximilians-Universität München, Munich, Germany; 2 Fraunhofer Institute for Translational Medicine and Pharmacology ITMP, Frankfurt, Germany

**Keywords:** Mechanism of action, Structure-based drug design, Biophysical chemistry

## Abstract

The neuroprotective transcription factor nuclear receptor related 1 (Nurr1, NR4A2) is in the focus of biomedical research for its promising neuroprotective role in Parkinson’s disease, Alzheimer’s disease, and multiple sclerosis. Its activity can be controlled by ligands offering access to pharmacological Nurr1 modulation. However, the binding epitope(s) and molecular activation mechanisms of synthetic Nurr1 activators remained elusive but are essential to advance Nurr1 ligands towards new medicines. Here we characterized Nurr1 dimer dissociation and coregulator release as molecular contributions to Nurr1 activation by vidofludimus and locate its binding in an allosteric surface pocket lined by helices 1, 5, 7, and 8 by mutagenesis and molecular dynamics simulation. Structure-guided ligand design using these insights resulted in an optimized Nurr1 agonist with substantially enhanced potency and binding affinity. Our results provide a structural and molecular basis for Nurr1 activation by a synthetic agonist which was lacking for rational ligand design.

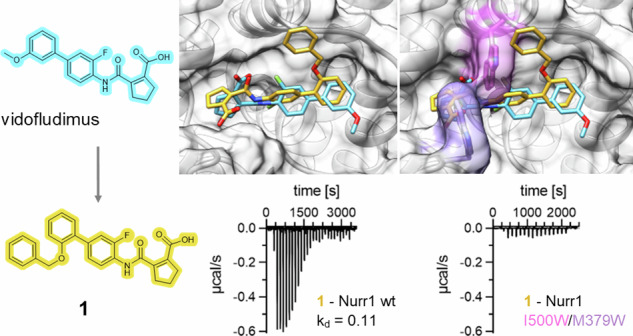

## Abstract (web summary)

Nuclear receptor related 1 (Nurr1) is a promising target for neuroprotection in diseases like Parkinson’s and Alzheimer’s, yet its activation mechanism by synthetic ligands remains unclear. Here, the authors elucidate Nurr1’s activation by vidofludimus, revealing its allosteric binding site and developing a more potent Nurr1 agonist, advancing rational drug design.

## Introduction

The ligand-sensing transcription factor nuclear receptor related 1 (Nurr1, NR4A2)^1^ is highly expressed in neurons and exhibits neuroprotective and anti-inflammatory activity^2,3^. Nurr1 plays an important role in regulating the expression of genes involved in dopamine metabolism, neurotransmission, axon development, mitochondrial function and transport, and cell survival^2,4,5^. Therefore, it is crucial for the differentiation, maturation, and maintenance of midbrain dopaminergic neuron clusters, making it an important factor in the pathology of neurodegenerative diseases^6–8^. Reduced concentrations of Nurr1 have been reported to increase susceptibility of dopaminergic neurons to damage induced by α-synuclein, which is a key factor implicated in Parkinson’s disease^8^. Additionally, reduced expression levels of Nurr1 have been correlated with progression of Alzheimer´s disease^9^. Due to the important role of Nurr1 in these pathologies, it is considered as a potential therapeutic target, and drugs that can activate Nurr1 may contribute to reduction of neuroinflammation and protection of dopaminergic neurons. However, potent Nurr1 agonists are rare, and structural and mechanistic understanding of Nurr1 modulation by ligands is limited.

Nurr1 belongs to the NR4A subgroup of nuclear receptors together with Nur77 (NR4A1)^10^ and NOR1 (NR4A3)^11^. All three receptors are highly conserved in their DNA-binding domain (DBD; 86–94% sequence identity) and ligand-binding domain (LBD; 66-69% sequence identity)^12^ and were initially considered as ligand-independent transcription factors^1^ since the canonical ligand binding region of nuclear receptors is blocked in NR4A receptors by tightly packed side chains of bulky hydrophobic residues^1^. This special structural characteristic leaves no space for ligand binding to the canonical pocket and stabilizes an autoactivated conformation with helix 12 bearing the activation function 2 (AF-2) bound to the core of the LBD resulting in high constitutive activity^13,14^. Still, all three NR4A receptors have been shown to respond to ligands^15–17^ with prostaglandin A1 and the oxidized dopamine metabolite 5,6-dihydroxyindole (DHI) considered as potential endogenous ligands of Nurr1. Both form a covalent adduct with Cys566 of the Nurr1 LBD^18,19^, and—together with an unpublished fragment ligand binding to Cys566 (PDB ID 8cyo)—are the only ligands successfully co-crystalized with Nurr1 to date (PDB ID 5y41, 5yd6, 6dda). The antimalarials amodiaquine (AQ) and chloroquine (CQ) were the first validated synthetic Nurr1 agonists^20^, and recently a few other drugs have been discovered as Nurr1 ligands including statins^21^ and the clinically studied dihydroorotate dehydrogenase (DHODH) inhibitor vidofludimus^22,23^. The latter exhibits sub-micromolar potency (EC_50_ 0.4 µM) and efficient agonism (310% activation) on Nurr1, thus emerging as a valuable tool to study Nurr1 biology^23,24^ but while direct and cellular target engagement of Nurr1 by vidofludimus has been broadly validated, the molecular and structural mechanisms of this activity remain elusive. Vidofludimus calcium showed beneficial effects in clinical trials for relapsing-remitting multiple sclerosis^22,25^ which could in part be due to Nurr1 agonism, thus advocating molecular elucidation of its effects on Nurr1.

Here we characterized the molecular activation mechanisms of Nurr1 by vidofludimus and explored the structural basis of its binding to Nurr1 by molecular dynamics simulation and mutagenesis. Compelling evidence from several orthogonal systems supported binding of the ligand in an allosteric surface pocket between helices 1, 5, 7, and 8. Structure-guided ligand design based on these findings yielded a Nurr1 agonist with enhanced potency and binding affinity.

## Results

### Molecular effects of vidofludimus on Nurr1 activity

**Fig. 1:**
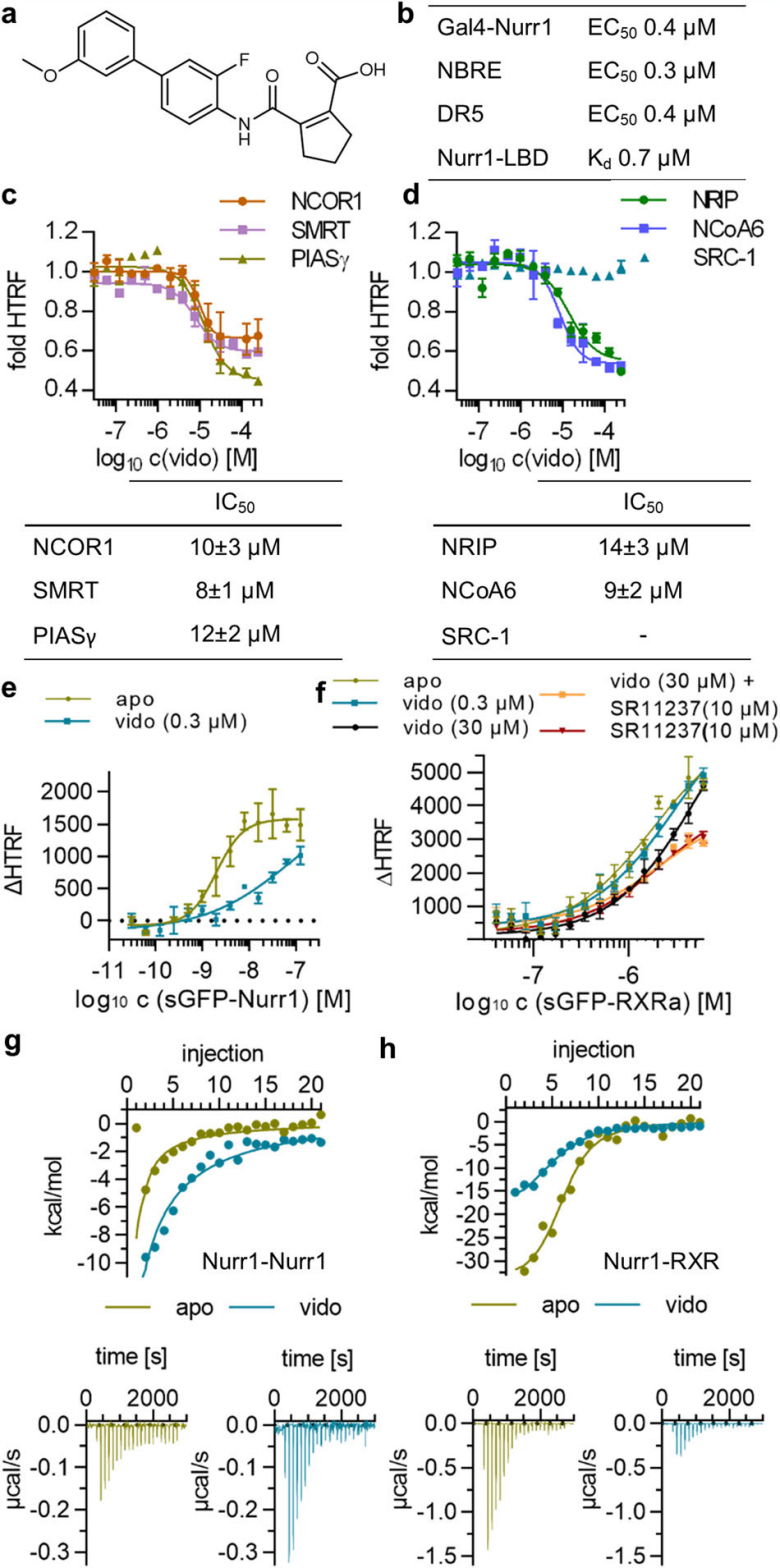
Molecular impact of vidofludimus (vido) on Nurr1 activity. **a** Chemical structure of vidofludimus. **b** Cellular potency of vidofludimus on Nurr1 and binding affinity to the Nurr1 LBD (from Vietor et.al.^23^). **c**, **d** Effects of vidofludimus on fluorescein labeled corepressor (**c**) and coactivator (**d**) binding to the Nurr1 LBD (Tb^3+^-cryptate labeled) in HTRF experiments. Data are the mean ± SD fold HTRF vs. DMSO ctrl; n = 3. Effects of vidofludimus on dimerization of Tb^3+^-cryptate labeled Nurr1 LBD with sGFP-labeled Nurr1 LBD (**e**) or RXRα LBD (**f**) in HTRF experiments. Data are the mean ± SD ΔHTRF vs. DMSO ctrl; n = 3. **g** Effect of vidofludimus on dissociation of the Nurr1 homodimer in ITC. 100 µM Nurr1 LBD was titrated to buffer in absence (apo) or presence of vidofludimus (2-fold excess) under otherwise identical conditions. **h** Effect of vidofludimus on Nurr1-RXR dimerization in ITC. 100 µM Nurr1 LBD was titrated to 15 µM RXRα LBD in absence (apo) or presence of vidofludimus (2-fold excess) under otherwise identical conditions.

Vidofludimus (Fig. 1a) was shown to activate Nurr1 in various cellular settings with sub-micromolar potency and to exhibit sub-micromolar binding affinity to the Nurr1 LBD (Fig. 1b). To explore how it modulates Nurr1 activity on the molecular level, we employed homogenous time-resolved fluorescence-resonance energy transfer (HTRF) based assays to determine ligand effects on the Nurr1 interactome which is crucial for activation. To study coactivator and corepressor binding, we employed Tb^3+^-cryptate labeled Nurr1 LBD as FRET donor and fluorescein labeled coregulator peptides as FRET acceptors. Nurr1 has been previously found to interact with the corepressors nuclear receptor corepressor 1 (NCOR1), silencing mediator of retinoid and thyroid receptors (SMRT), protein inhibitor of activated STAT protein gamma (PIASγ), and REST corepressor 1 (CoREST) as well as the coactivators nuclear receptor-interacting protein 1 (NRIP) and nuclear receptor coactivator 6 (NCoA6)^26–28^. Additionally, we evaluated recruitment of the proposed Nurr1 interacting protein LIM homeodomain transcription factor 1b (Lmx1b)^29^. Lmx1b bound to Nurr1 but showed no response to vidofludimus, the effect of the ligand on CoREST binding was weak, and the steroid receptor coactivator 1 (SRC-1) neither bound to ligand-free Nurr1 nor in presence of vidofludimus. However, NCOR1, SMRT, PIASγ, NRIP, and NCoA6 were robustly displaced from the Nurr1 LBD by vidofludimus demonstrating that the ligand strongly affected Nurr1-coregulator interactions (Fig. 1c,d and Supplementary Figs. 1, 2).

Apart from coregulator interactions which mediate recruitment of chromatin-modifying enzymes and the transcriptional machinery, dimerization is another key mechanism of nuclear receptor regulation^30,31^. Previous studies^32,33^ suggested that heterodimerization diminishes the activity of Nurr1 and that ligand-induced dissociation of the transcriptionally inactive Nurr1-RXR heterodimer mediates Nurr1 activation. This was also evident from HTRF experiments observing the dimerization of the Tb^3+^-cryptate labeled Nurr1 LBD and the sGFP-labeled Nurr1 LBD or RXRα LBD. Vidofludimus strongly reduced Nurr1 homodimerization (Fig. 1e) and also destabilized the Nurr1-RXR heterodimer (Fig. 1f) but to a lesser extent. Similar results were obtained in isothermal titration calorimetry (ITC) experiments where vidofludimus markedly enhanced dissociation of the Nurr1 homodimer when the protein was titrated into buffer (Fig. 1g, Supplementary Tab. 1) and reduced Nurr1-RXR dimerization when Nurr1 was titrated to RXR (Fig. 1h, Supplementary Tab. 1).

These results thus indicated that Nurr1 activation by vidofludimus involves several molecular contributions among which diminished Nurr1 homodimer stability emerged as strongest effect. Coregulator displacement, which occurred with an order of magnitude lower potency (Fig. 1c, d) compared to the affinity and cellular EC_50_ of vidofludimus, may hence be a secondary effect of altered dimerization.

### Evaluation of the vidofludimus-Nurr1 binding epitope

**Fig. 2:**
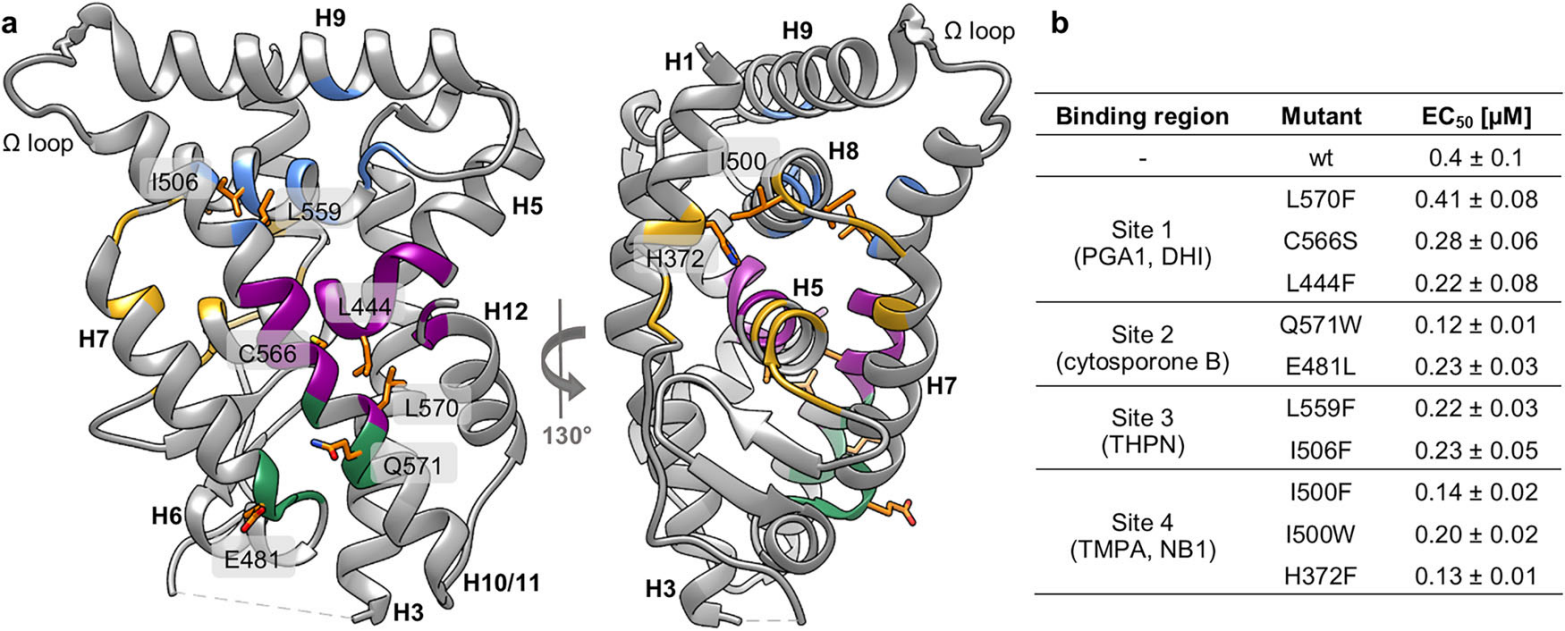
Proposed ligand binding regions in the Nurr1 LBD, residues selected for mutagenesis, and effects of mutations on the potency of vidofludimus. **a** The binding region of prostaglandin A1 (PDB ID: 5y41^18^) and DHI (PDB ID: 6dda^19^) was mapped on the apo-Nurr1 structure (PDB ID: 1ovl^1^) in purple. Ligand binding sites in the Nur77 LBD were mapped on the apo-Nurr1 structure (PDB ID: 1ovl^1^) based on Nur77 bound to cytosporone B (PDB ID: 6kz5^37^) in green, THPN (PDB ID: 4jgv^35^) in cyan and TMPA and NB1 (PDB ID: 3v3q^36^ and 8y7l^34^, respectively) in purple and yellow. Residues selected for site-directed mutagenesis are labeled and shown in orange. **b** EC_50_ values of vidofludimus on wt Gal4-Nurr1 and mutants. Activation of wt Nurr1 and mutants was determined in uniform Gal4 hybrid reporter gene assays. Data are the mean ± S.E.M., n = 3. Activity data on wt Nurr1 have been reported previously^23^.

After profiling the Nurr1 activation mechanisms of vidofludimus, we set out to explore the drug’s molecular interaction with the transcription factor. As crystal structures of ligand-bound Nurr1 have only been solved for covalent binders to date, we aimed to study the interaction by modeling and mutagenesis. Crystallography of the close relative Nur77 of Nurr1 has been more successful^34–37^ offering a template to identify potential ligand binding regions. Based on alignment of Nurr1 and Nur77, we proposed four possible binding epitopes and designed ten mutants (Fig. 2) for initial screening. Hybrid Gal4-Nurr1 mutants were then generated by site-directed mutagenesis to compare the activity of vidofludimus on these mutants with its effect on wild-type (wt) Nurr1 in reporter gene assays. The assays were conducted in a uniform format using a Gal4-responsive firefly luciferase reporter construct and a constitutively expressed (SV40 promoter) renilla luciferase as control gene for normalization. All mutant Gal4-Nurr1 clones were functional and exhibited constitutive activity like the wt hybrid receptor (Supplementary Figure 3).

Vidofludimus activated all ten tested mutant Gal4-Nurr1 receptors with sub-micromolar potency (Fig. 2b) indicating that none of the mutations prevented binding. The EC_50_ values of the drug on Q571W, I506F, I500F, and H372 were lower than on the wt receptor. Q571 (site 2) is located at the dimer interface and mutation of this residue might affect dimerization of Nurr1 which in turn could modulate its response to ligands. I506 (site 3) neighbors a SUMOylation site which could also be affected by mutation and thus alter the response to ligands. Effects of the Q571W and I506F mutations on the activity of vidofludimus could hence not be confidently referred to altered ligand binding, especially since mutagenesis of the related residues E481 and L559 in the same sites had less effect. I500F and H372F (both site 4), in contrast, had a more consistent impact on Nurr1 modulation by vidofludimus providing preliminary support for interaction of the drug with site 4 in the Nurr1 LBD. Importantly, the I500F and H372F mutations did not alter Nurr1 activation by the agonist fluvastatin (Supplementary Fig. 4) indicating their effect as specific for vidofludimus.

**Fig. 3:**
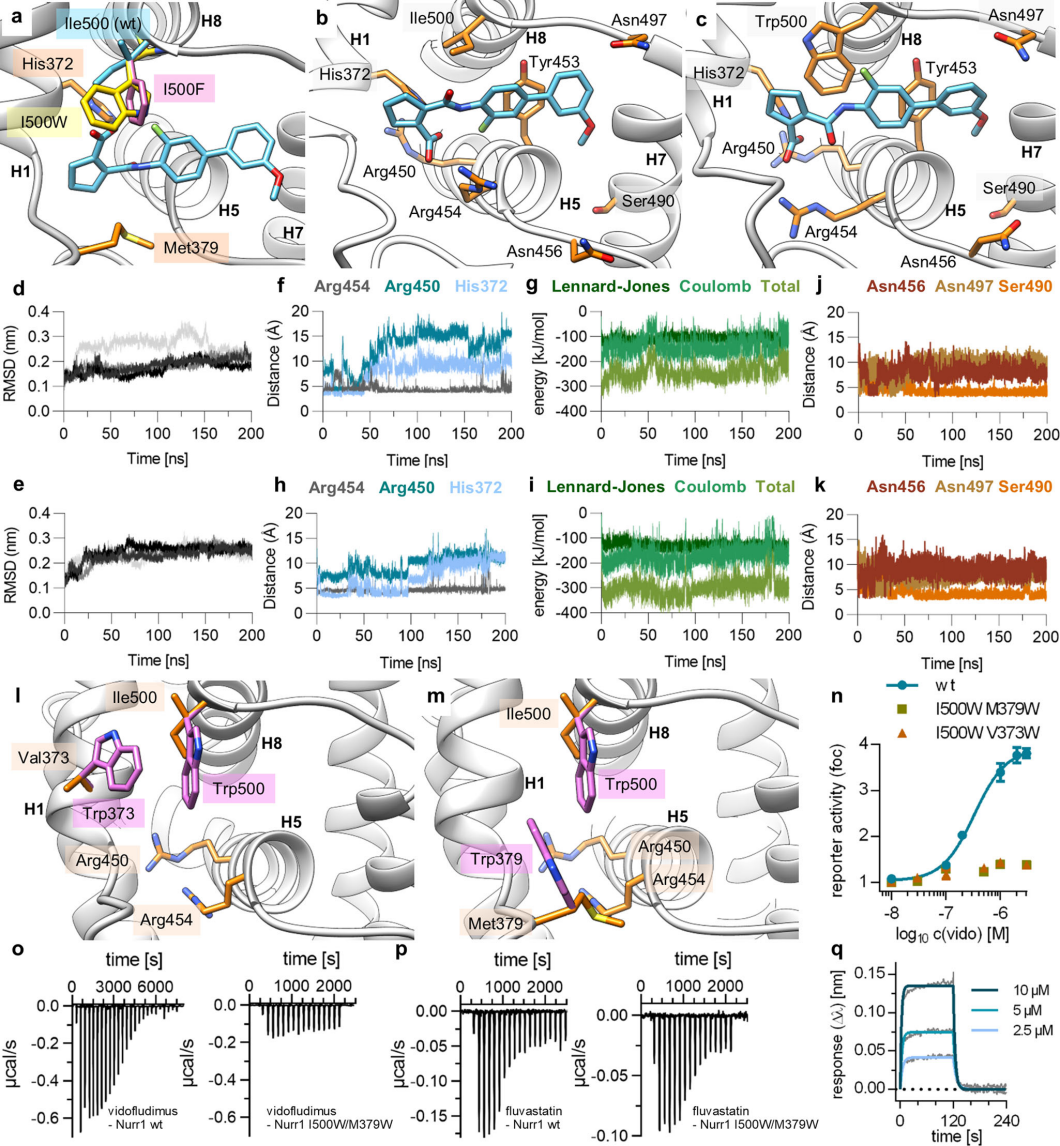
Binding of vidofludimus (vido) to the Nurr1 LBD. **a** Docking of vidofludimus (blue) to site 4 with the activity modulating mutants I500F (pink) and I500W (yellow) highlighted. Both mutations potentially enable an additional π-interaction. Other relevant residues are shown in orange. **b** Molecular dynamics (MD) simulation (wt Nurr1 is shown) suggested a rapid flip of the cyclopentenecarboxylic acid motif of vidofludimus (blue) towards His372/Arg450/Arg454 inducing a transient pocket with enhanced polar interactions. The complex with the strongest ligand binding energy is shown, and relevant residues are shown in orange. **c** MD simulation of Nurr1-I500W indicated additional interactions of vidofludimus (blue) with Trp500 and a shifted binding mode with stronger contacts to Arg454. The complex with the strongest ligand binding energy is shown, relevant residues are shown in orange. RMSD for the wt Nurr1-vidofludimus complex (**d**) and Nurr1-I500W-vidofludimus complex (**e**) in 200 ns MD simulations (four repeats are shown). **f** Distances of the ligand carboxyl-C to His372, Arg450, and Arg454 in the wt Nurr1-vidofludimus complex simulation. **g** Ligand binding energies of the wt Nurr1-vidofludimus complex simulation. **h** Distances of the ligand carboxyl-C to His372, Arg450, and Arg454 in the Nurr1-I500W-vidofludimus complex simulation. **i** Ligand binding energies of the Nurr1-I500W-vidofludimus complex simulation. **j** Distances of the ligand methoxy-CH_3_ to Asn456, Asn497 and Ser490 in the wt Nurr1-vidofludimus complex simulation. **k** Distances of the ligand methoxy-CH_3_ to Asn456, Asn497 and Ser490 in the Nurr1-I500W-vidofludimus complex simulation. **l**, **m** The double mutations I500W/V373W (pink) and I500W/M379W (pink) of the Nurr1 LBD block the proposed vidofludimus binding site, relevant residues of Nurr1-wt are shown in orange. **n** Vidofludimus failed to activate the double Nurr1 mutants I500W/V373W and I500W/M379W. Data are the mean ± S.E.M. fold reporter activation vs. DMSO control from Gal4-Nurr1 hybrid reporter gene assays; n = 3. **o** Isothermal titration calorimetry (ITC) showed high-affinity binding of vidofludimus to the Nurr1 wt LBD (K_d_ 0.7 µM; from Vietor et al.^23^) but no or very weak binding to the Nurr1 I500W/M379W mutant LBD. The isotherms of the compound-protein titrations are shown. **p** ITC showed high-affinity binding of fluvastatin to the Nurr1 wt LBD (K_d_ 0.6 µM) and to the Nurr1 I500W/M379W mutant LBD (K_d_ 0.5 µM). The isotherms of the compound-protein titrations are shown. Thermodynamic parameters are shown in Supplementary Tab. 2. **q** Binding of vidofludimus to the Nurr1 LBD (wt) in biolayer interferometry (BLI) revealing a fast off-rate (k_off_ 0.233 s⁻¹).

The mutant-derived binding site hypothesis was further supported by modeling the interaction of vidofludimus with the proposed epitope in the Nurr1 LBD. Docking suggested that site 4 could accomodate the drug well with several hydrophobic ligand-protein contacts and H-bonds involving His372 and the backbone of Met379 (Fig. 3a). In silico mutation to I500F, which enhanced potency of vidofludimus in vitro, enabled an additional π-interaction thus providing preliminary explanation for the altered activity. Enhanced potency of vidofludimus was retained on a corresponding Nurr1-I500W mutant in vitro (EC_50_ 0.20 ± 0.02 µM) supporting the hypothesis of an additional π-contact.

To further interrogate the proposed binding of vidofludimus at site 4 and how the binding site mutations could enhance affinity, we performed molecular dynamics (MD) simulations^38^ (200 ns) for the wt Nurr1 LBD (Fig. 3b, Supplementary Data 1) and the I500W mutant (Fig. 3c, Supplementary Data 1) starting from the top-scoring docking pose of bound vidofludimus. Both complexes revealed high stability with low RMSD over the entire simulation (Fig. 3d, e). The ligand’s cyclopentene motif flipped within < 50 ps to expose the carboxylate towards a triad of basic side chains (His372, Arg450 and Arg454) enabling an additional ionic contact (Fig. 3b). The wt Nurr1 simulation suggested two binding modes with the carboxylate interacting with His372 and flipping between contacts to Arg450 or Arg454. The Arg454 interaction dominated and was stable after 50 ns simulation (Fig. 3f), despite similar ligand binding energies of both poses (Fig. 3g). The Nurr1-I500W simulation also revealed preferential interaction of the ligand’s carboxylic acid with Arg454 but lower distances to Arg450 and His372 (Fig. 3h), and, accordingly, the ligand binding energy was slightly stronger in the mutant complex (Fig. 3i). These observations thus indicated that the I500W mutation stabilized the ligand’s preferred binding mode explaining the increased potency observed in vitro. The terminal methoxyphenyl residue of vidofludimus bound between Asn456 and Asn497 in both complexes with rather stable orientation of the methoxy group towards Ser490 (Fig. 3j, k).

Next, we used additional mutants designed to prevent vidofludimus binding to obtain further evidence for binding of the ligand to site 4 as proposed by mutagenesis and MD simulation. We employed the double mutations I500W/M379W and I500W/V373W introducing bulky side chains to block the binding site (Fig. 3l, m, Supplementary Fig. 5). Both mutants were functional and responded to the agonist fluvastatin (Supplementary Fig. 4) but led to a complete loss of activation by vidofludimus (Fig. 3n). Vidofludimus also showed no binding to the recombinantly expressed Nurr1-I500W/M379W double mutant LBD protein in ITC (Fig. 3o) while affinity of fluvastatin was not altered by the mutations (Fig. 3p). Additionally, the proposed binding of vidofludimus to a shallow surface epitope was also supported by its binding properties in biolayer interferometry (BLI) which revealed a fast dissociation rate (k_off_ 0.233 s⁻¹; Fig. 3q).

These results from mutagenesis, binding studies, and modeling supported our hypothesis of vidofludimus interacting with site 4 of the Nurr1 LBD. Modeling further suggested that the carboxylate motif of the drug interacts with a triad (Arg454, Arg450, and His372) of basic side chains indicating importance of this polar ligand feature. Moreover, the central aromatic part of the ligand was bound according to the model in a rather narrow groove which aligns with our previous observations that larger substituents on this ring and its replacement by bulky bicyclo systems are not tolerated^23^. The experimentally determined SAR of vidofludimus as Nurr1 ligand^23^ thus aligned with the proposed Nurr1-vidofludimus interaction model, too.

Introduction of aromatic residues in the central part of the pocket (I500F, I500W) enhanced agonist potency of vidofludimus in vitro possibly by offering an additional π-contact (Fig. 3c). According to MD, the bulkier aromatic I500W induced a slightly flipped and stabilized binding of the central ligand scaffold which strengthened the ionic interaction with Arg454 leading to higher stability of the complex. Enhanced potency of vidofludimus on the H372F mutant is likely due to a similarly enhanced neutralizing contact to Arg454/Arg450. The double mutants I500W/M379W and I500W/V373W, blocking the pocket and disrupting the binding of vidofludimus, contribute further to experimental validation of our Nurr1-vidofludimus interaction model.

### Structure-guided design of a potent vidofludimus analog

**Fig. 4:**
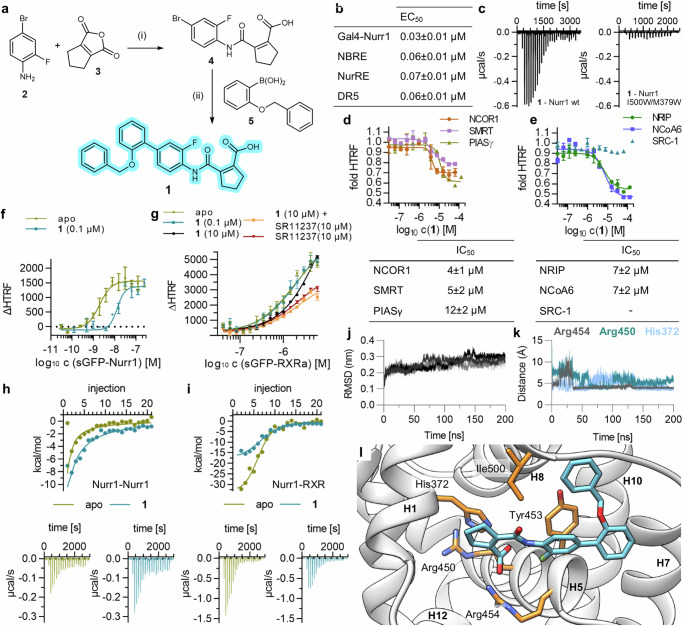
Structure guided design enables development of the optimized vidofludimus analog 1. **a** Synthesis scheme of **1**. Reagents & Conditions: (i) CH_2_Cl_2_, rt, 16 h, 99%; (ii) XPhos-Pd-G2, Cs_2_CO_3_, PhCH_3_/EtOH/H_2_O 3:2:1, 100 °C, 18 h, 65%. **b** Activities of **1** in cellular reporter gene assays for Gal4-Nurr1 and full-length Nurr1 on the monomer (NBRE), homodimer (NurRE), and RXR-heterodimer (DR5) response elements. Data are the mean ± S.E.M., n = 3. **c** Isothermal titration calorimetry (ITC) showed high-affinity binding of **1** to the Nurr1 wt LBD (K_d_ 0.11 µM) but no binding to the Nurr1 I500W/M379W mutant LBD. The isotherms of the compound-protein titrations are shown. Thermodynamic parameters are shown in Supplementary Tab. 2. **d** Effects of **1** on corepressor (fluorescein labeled) binding to the Nurr1 LBD (Tb^3+^-cryptate labeled) in HTRF experiments. Data are the mean ± SD fold HTRF vs. DMSO ctrl; n = 3. **e** Effects of **1** on coactivator (fluorescein labeled) binding to the Nurr1 LBD (Tb^3+^-cryptate labeled) in HTRF experiments. Data are the mean ± SD fold HTRF vs. DMSO ctrl; n = 3. **f**, **g** Effects of **1** on dimerization of Tb^3+^-cryptate labeled Nurr1 LBD and sGFP-labeled Nurr1 LBD (**f**) or RXRα LBD (**g**) in HTRF experiments. Data are the mean ± SD ΔHTRF; n = 3. **h** Effect of **1** on dissociation of the Nurr1 homodimer in ITC. 100 µM Nurr1 LBD was titrated to buffer in absence (apo) or presence of **1** (2-fold excess) under otherwise identical conditions. **i** Effect of **1** on Nurr1-RXR dimerization in ITC. 100 µM Nurr1 LBD was titrated to 15 µM RXRα LBD in absence (apo) or presence of **1** (2-fold excess) under otherwise identical conditions. **j** The Nurr1-**1** complex was stable over 200 ns MD simulation with low RMSD (four repeats). **k** Distances of the carboxyl-C of **1** to His372, Arg450 and Arg454 in the wt Nurr1-**1** complex simulation. **l** Binding mode of **1** (blue) to the Nurr1 LBD in the complex with the strongest ligand binding energy in MD simulation, relevant residues are shown in orange.

Using these valuable structural insights into the interaction of vidofludimus with Nurr1 to improve agonist potency, we designed and synthesized an optimized analog. The proposed binding mode revealed an ample surface pocket close to the ortho-position of the terminal methoxyphenyl motif that could be explored with an additional substituent (Fig. 3b). Hence, we removed the methoxy group of vidofludimus and introduced a benzyloxy residue in the promising ortho position in compound **1**. The designed ligand **1** was synthesized over two steps by reaction of aniline **2** with the anhydride **3** to the amide **4** and subsequent Suzuki coupling with boronic acid **5** with good overall yield (Fig. 4a). In vitro testing of **1** revealed strong Nurr1 agonism with 10-fold higher potency on Gal4-Nurr1 than vidofludimus (Fig. 4b) and 7-fold enhanced affinity (Fig. 4c). This substantial improvement in Nurr1 agonism further corroborated the proposed binding mode which served as design hypothesis. Full cellular profiling of **1** also demonstrated potent activation of full-length human Nurr1 on the monomer (NBRE), homodimer (NurRE), and RXR-heterodimer (DR5) response elements (Fig. 4b), and the molecular effects of **1** on coregulator binding to the Nurr1 LBD resembled the parent drug vidofludimus with slightly higher potency of **1** (Fig. 4d, e). Moreover, as observed for vidofludimus, presence of **1** had a strong impact on dimerization of Nurr1 in HTRF (Fig. 4f, g) and ITC (Fig. 4h, i) experiments. **1** markedly disrupted the Nurr1 homodimer and weakened Nurr1-RXR dimerization further indicating that release of the – likely more transcriptionally active – Nurr1 monomer contributed to the molecular activation mechanism by this agonist scaffold.

The binding kinetics of **1** displayed a slower off-rate (k_off_ 0.0224 s⁻¹) compared to vidofludimus corroborating the improved affinity of **1** (Supplementary Fig. 6). MD simulation of the Nurr1-**1** complex (Fig. 4j–l, Supplementary Data 1) supported binding of **1** to the vidofludimus binding site. As observed for vidofludimus, the complex was stable with low RMSD over the entire simulation and revealed strong interactions of the ligand’s carboxylic acid with the triad of basic residues (His372, Arg450, Arg454). Lower distances of the carboxylate of **1** to Arg450 and His372 compared to vidofludimus and additional contacts of the benzyloxy motif of **1** with hydrophobic regions of the Nurr1 LBD surface rationalized the ligand’s higher affinity and in vitro potency. Like vidofludimus, **1** displayed no binding to the Nurr1-I500W/M379W double mutant LBD in ITC (Fig. 4c), experimentally supporting the proposed binding site.

The Nurr1-vidofludimus interaction model thus enabled structure-informed analog design of the functionally similar but more potent derivative **1** corroborating the potential of the model for further Nurr1 ligand development based on vidofludimus and possibly other scaffolds.

## Discussion

Beneficial effects of Nurr1 activators in models of Parkinson’s disease, Alzheimer’s disease, and multiple sclerosis^20,39–42^ suggest pharmacological Nurr1 activation as an emerging approach to new therapies in neurodegeneration, and considerable progress has been made in the discovery of Nurr1 ligands as chemical tools and leads^23,39,40,43,44^. However, molecular understanding of Nurr1 modulation by ligands is still limited. Cocrystal structures have revealed covalent binding of the endogenous ligands DHI and PGA1 to a pocket behind helix 12^18,19^ and NMR-based studies support binding of unsaturated fatty acids and the antimalarial amodiaquine to a surface region of the Nurr1 LBD^27,45^. The binding epitopes of potent synthetic Nurr1 agonists remained elusive.

Here we developed a model for binding of the Nurr1 agonist vidofludimus at an allosteric surface pocket lined by helices 1, 5, 7, and 8, and distant from the canonical activation function in helix 12. Results from mutagenesis, cellular, and biophysical assays provided compelling evidence for this proposed Nurr1-ligand interaction. The considerable potency and binding affinity of vidofludimus and the optimized analog **1** indicate that the pocket is druggable and may offer access to other Nurr1 modulator scaffolds through structure-guided design. Further ligand optimization should consider the fast off-rate of vidofludimus and **1** to achieve stronger Nurr1 agonism.

Disruption of Nurr1 dimerization by vidofludimus and **1** emerged as strong and possibly key molecular contribution to the activation mechanism supporting the hypothesis that the Nurr1 monomer is the receptor’s transcriptionally most active form^32^. Additionally, both ligands modulated the recruitment of coregulators to the Nurr1 LBD as further mechanistic component. The observed ligand-induced displacement of all studied co-regulators might reflect a secondary effect of reduced Nurr1 homodimerization. However, we only studied a subset of possible Nurr1 interactors and other coregulators of Nurr1 may remain to be identified that are recruited upon vidofludimus binding.

These molecular insights into Nurr1 modulation by vidofludimus as one of the most active known agonists, substantially advance structural and mechanistic understanding of the transcription factor and will aid future ligand discovery to enable Nurr1-based therapies in neurodegeneration.

## Methods

### In vitro assays

#### Site-directed mutagenesis

Mutations in the Nurr1 LBD (L570F, C566S, L444F, Q571W, E481L, L559F, I506F, I500F, I500W or H372F and combinations of I500W and V373W or M379W) were introduced by site-directed mutagenesis using pairs of forward and reverse oriented primers containing the modified positions (primer sequences are reported in Supplementary Tab. 3). Mutations L570F, C566S, L444F, Q571W, E481L, L559F, I559F, I506F and I500F were introduced using the Phusion Site-Directed Mutagenesis Kit (Thermo Scientific) following the manufacturer's protocol. Mutations I500W, H372F, V373W, and M379W were introduced using the QuickChange II Site Directed Mutagenesis Kit (Agilent). Introduction of the desired mutation and integrity of the Gal4-hNurr1 open reading frame was verified by direct sequencing (Sanger).

#### Reporter gene assays

Modulation of wt and mutant Nurr1 activity by ligands was determined in Gal4 hybrid reporter gene assays^46^ in transiently transfected HEK293T cells (German Collection of Microorganisms and Cell Culture GmbH, DSMZ) using pFA-CMV-hNurr1-LBD^26^ or a respective mutant coding for the Gal4-DBD fused to the hinge region and ligand binding domain of the canonical isoform of human Nurr1. pFR-Luc (Stratagene, La Jolla, CA, USA) served as Gal4 responsive reporter, and pRL-SV40 (Promega, Madison, WI, USA) was used as internal control to monitor transfection efficiency and test compound toxicity. Modulation of full-length human Nurr1 was studied using the reporter plasmids pFR-Luc-NBRE^26^, pFR-Luc-NurRE^26^ or pFR-Luc-DR5^26^, the full-length human nuclear receptor Nurr1 encoded by pcDNA3.1hNurr1-NE (Addgene plasmid #102363), the full length human RXR encoded by pSG5-hRXR and pRL-SV40. Cells were cultured in Dulbecco’s modified Eagle’s medium (DMEM), high glucose supplemented with 10% fetal calf serum (FCS), sodium pyruvate (1 mM), penicillin (100 U/mL), and streptomycin (100 μg/ mL) at 37 °C and 5% CO_2_ and seeded in 96-well plates (3 × 10^4^ cells/well). After 24 h, the medium was changed to Opti-MEM without supplements, and the cells were transiently transfected using the Lipofectamine LTX reagent (Invitrogen) according to the manufacturer’s protocol. Five hours after transfection, the cells were incubated with the test compounds in Opti-MEM supplemented with penicillin (100 U/mL), streptomycin (100 μg/mL), and 0.1% DMSO for 14-16 h before luciferase activity was measured using the Dual-Glo Luciferase Assay System (Promega) according to the manufacturer’s protocol on a Tecan Spark luminometer (Tecan Deutschland GmbH, Germany). Firefly luminescence was divided by Renilla luminescence and multiplied by 1000, resulting in relative light units (RLUs) to normalize for transfection efficiency and cell growth. Fold activation was obtained by dividing the mean RLU of a test sample by the mean RLU of the untreated control. All samples were tested in at least three biologically independent experiments in duplicates. For dose-response curve fitting and calculation of EC_50_ values, the equation “[Agonist] versus response–variable slope (four parameters)” was used in GraphPad Prism (version 9.5.1, GraphPad Software, La Jolla, CA).

#### Recombinant Expression and Purification of Nurr1 LBD fusion Protein

For generation of biotinylated Nurr1 a plasmid construct encoding Nurr1 (aa 364-598) in a pMal vector system (New England Biolabs, NEB, Ipswich, MA, USA) was used^26^. For protein expression, *E.coli* BL21-R3-pRARE2 cells (Thermo Fisher) were transformed with the Nurr1 expression construct and selected overnight on LB (Luria Broth) containing 34 µg/ml chloramphenicol and 100 µg/ml ampicillin. Culture in liquid Terrific Broth (TB) was inoculated and grown at 37 °C with constant shaking at 180 rpm until optical density at 600 nm (OD_600_) reached 0.5. At this time point, the temperature was reduced to 18 °C and shaking to 120 rpm. Approximately 1 h later OD_600_ reached 0.9, and expression of the target protein was induced by addition of 0.5 mM IPTG. After 16 h, the culture was harvested by centrifugation at 5000 × *g* at 4 °C for 20 min. Cell pellets corresponding to 2 liters of culture were resuspended in 100 ml of buffer A (400 mM NaCl, 20 mM NaPi pH 7.8, 10% (w/v) glycerol, and 20 mM ß-mercaptoethanol). Cells were kept on ice and disrupted by sonication in the presence of 1 mM ATP, DNAse I, 10 mM MgSO_4_, EDTA-free cOmplete™ protease inhibitor cocktail (F. Hoffmann-La Roche AG, Basel, Switzerland), and lysozyme. Sonication was performed in cycles of 5 s on/10 s off at 35% for 18 min. Nucleic acids were precipitated by adding PEI 0.15%. Cell debris was removed by centrifugation at 5000 × *g* for 30 min at 4 °C and at 40,000 × *g* for 30 min at 4 °C. Purification of the target protein was achieved by immobilized metal affinity chromatography (IMAC) using columns packed with Ni Sepharose 6 Fast Flow resin on an ÄKTApurifier FPLC system (GE Healthcare, Chicago, IL, USA). After washing with buffer A supplemented with 10 mM imidazole the protein was eluted with 50 mM imidazole. Afterwards, the fusion protein was processed with His tagged TEV protease overnight, and imidazole content was reduced to 10 mM by dialysis against buffer A. Afterwards, uncleaved fusion protein, free MBP-Tag, and TEV were removed by reverse IMAC. The flow-through from reverse IMAC containing Nurr1 LBD was collected and supplemented with 0.5 mM biotin, 0.5 mM ATP, 5 mM MgCl_2_, and *E.coli* biotin ligase BirA at a molar ratio of ~1:10 for enzymatic conjugation of biotin to the lysine residue in the avitag. After overnight incubation at 4°C, the solution was subjected to reverse IMAC to remove biotin ligase birA. The product was concentrated and subjected to size exclusion chromatography using 20/30 Superdex75^TM^ column equilibrated and run in HTRF buffer (25 mM HEPES pH 7.5, 10% (m/v) glycerol, 5 mM DTT).

#### Recombinant expression and purification of Nurr1-I500W/M379W LBD protein

*E.coli*-BL21(DE3) cells (Thermo Fisher) were transformed with a pET24a(+) based expression plasmid coding for the I500W/M379W double mutant Nurr1-LBD (GenScript), and selected overnight in liquid Terrific Broth (TB) containing 35 µg/ml Kanamycin. Culture in TB was inoculated and grown at 37 °C with constant shaking at 180 rpm until optical density at 600 nm (OD_600_) reached 0.5. At this time point, the temperature was reduced to 18 °C and shaking to 120 rpm. The target protein was induced by addition of 0.5 mM IPTG. After 16 h, the culture was harvested by centrifugation at 5000 × *g* at 4 °C for 20 min. Cell pellets corresponding to 2 liters of culture were resuspended in 100 ml of buffer B (500 mM NaCl, 50 Mm Tris pH 7.8, 5% (w/v) glycerol, and 0.5 mM TCEP mM) supplemented with 25 mM imidazole. Cells were kept on ice and disrupted by sonication in the presence of EDTA-free cOmplete™ protease inhibitor cocktail (F. Hoffmann-La Roche AG, Basel, Switzerland). Sonication was performed in cycles of 5 s on/10 s off at 35% for 18 min. Cell debris was removed by centrifugation at 5000 x g for 30 minutes at 4°C and at 40,000 × *g* for 30 min at 4 °C. Purification of the target protein was achieved by IMAC using columns packed with Ni Sepharose 6 Fast Flow resin. After washing with buffer B supplemented with 25 mM imidazole the protein was eluted with 300 mM imidazole. Afterwards, the fusion protein was processed with His tagged TEV protease overnight, and imidazole content was reduced to 10 mM by dialysis against buffer B. Uncleaved fusion protein, free His-Tag, and TEV were removed by reverse IMAC. The flow-through from reverse IMAC containing the mutant Nurr1 LBD was collected and subjected to size exclusion chromatography using 20/30 Superdex75^TM^ column equilibrated and run in buffer B. The purified protein was dialyzed against ITC buffer and concentrated.

#### Nurr1 co-regulator recruitment assay

Homogeneous time-resolved fluorescence energy transfer (HTRF) experiments were used to study the recruitment of co-regulator peptides to the Nurr1-LBD in the presence or absence of test compounds. Terbium cryptate as streptavidin conjugate (Tb-SA; Cisbio Bioassays, Codolet, France) was used as FRET donor for stable coupling to biotinylated recombinant Nurr1 LBD protein. Fluorescein-labeled co-regulator peptides were used as FRET acceptors (peptide sequences are reported in Supplementary Tab. 4). Assay solutions were prepared in HTRF assay buffer supplemented with 0.1% (w/v) CHAPS and contained recombinant biotinylated Nurr1 LBD (final concentration 1.5 nM), Tb-SA (1.5 nM) and the respective fluorescein-labeled co-regulator peptide (100 nM) as well as 1% DMSO with the test compounds at varying concentrations for dose–response experiments. Recruitment of CoREST and Lmx1b peptides was studied using biotin-labeled peptides for stable coupling to Tb-SA as FRET donor and sGFP-Nurr1^26^ LBD as FRET acceptor. Assay solutions were prepared in HTRF assay buffer supplemented with 0.1% (w/v) CHAPS and contained Tb-SA (3 nM), biotin-labeled peptide (3 nM), and 1% DMSO with or without vidofludimus (3 or 30 µM) or **1** (1 or 10 µM), while sGFP-Nurr1 LBD was titrated from 0.5 µM, and free sGFP was added to keep the total GFP content stable at 0.5 μM to suppress artefacts from diffusion enhanced FRET. Additionally, compound effects on CoREST were studied by titrating vidofludimus, **1**, or fluvastatin (highest concentration 100 µM) in presence of fixed concentrations of biotinylated CoREST peptide (3 nM) coupled to Tb-SA (3 nM) and sGFP-Nurr1 LBD (100 nM) in HTRF buffer. Experiments were carried out in 384 well format using white flat bottom small volume polystyrol microtiter plates (Greiner Bio-One, Frickenhausen, Germany). After 2 h incubation at rt, fluorescence intensities (FI) after excitation at 340 nm were recorded at 520 nm for fluorescein acceptor fluorescence and 620 nm for Tb-SA donor fluorescence on a SPARK plate reader (Tecan Group Ltd.). FI520 nm was divided by FI620 nm and multiplied with 10,000 to give a dimensionless HTRF signal. For dose-response curve fitting, the equation “[Inhibitor] versus response–variable slope (four parameters)” was used in GraphPad Prism (version 9.5.1, GraphPad Software, La Jolla, CA).

#### Nurr1 dimer formation assays

Modulation of Nurr1 LBD homodimerization and Nurr1-RXRα heterodimerization by ligands was studied by HTRF. sGFP-Nurr1 LBD protein and sGFP-RXRα LBD^26^ were titrated against a fixed concentration of Tb-SA conjugated Nurr1 LBD protein. Assay solutions were prepared in HTRF assay buffer supplemented with 0.1% (w/v) CHAPS as well as 1% DMSO with test compounds at fixed concentrations or DMSO alone as negative control (apo). The FRET donor complex formed from biotinylated Nurr1 LBD (final concentration 0.188 nM) and Tb-SA (0.375 nM) was kept constant while the concentration of sGFP-labeled protein was varied. For the heterodimerization assay, sGFP-RXRα LBD concentration was titrated from 6 µM, while free sGFP was added to keep the total GFP content stable at 6 μM. For the homodimerization assay, sGFP-Nurr1 LBD protein was titrated from 0.5 µM, while free sGFP was added to keep the total GFP content stable at 0.5 μM. Samples were equilibrated at RT for 2 h before FI520 and FI620 were recorded after excitation at 340 nm, and the HTRF signal was calculated as described above. For dose-response curve fitting, the equation “[Agonist] versus response–variable slope (four parameters)” was used in GraphPad Prism (version 9.5.1, GraphPad Software, La Jolla, CA).

#### Binding kinetics evaluation by biolayer interferometry (BLI)

BLI measurements were conducted using the Octet R8 instrument (Sartorius). Nurr1 was enzymatically biotinylated using BirA, and Super Streptavidin (SSA) sensors were used to capture the biotinylated Nurr1. Prior to the assay, SSA sensors were pre-soaked for 10 minutes in a buffer containing 20 mM HEPES (pH 7.4), 150 mM NaCl, 0.01% Tween-20, and 0.5% DMSO. The sensors were then loaded with 2 μM biotinylated Nurr1 for 10 min, reaching a response of ~10 nm. Free biotin sites were blocked by incubating the sensors with 20 μg/ml biocytin for 1 minute. After quenching, the sensors were washed for 20 min with HEPES buffer and equilibrated for 5 min in the same buffer supplemented with 1% PEG3K. The binding of compounds was monitored for 120 s, followed by a 120 s dissociation phase. Two control experiments were conducted: one with sensors coated with the protein but without the compound, and the other with uncoated biosensors exposed to the compound at corresponding concentrations. These controls were used to correct for non-specific binding and baseline interference.

#### Isothermal titration calorimetry

ITC experiments were conducted on an Affinity ITC instrument (TA Instruments, New Castle, DE) using Nurr1 LBD^24^ protein and RXRα LBD^47^ protein. Results were analyzed using NanoAnalyze software (version 3.11.0, TA Instruments, New Castle, DE) with independent binding models unless otherwise stated. All ITC experiments were performed at least twice (N = 2). The binding affinity of **1** and fluvastatin to the Nurr1 LBD were determined by titrating Nurr1 LBD (5–10 µM) in buffer (20 mM Tris pH 7.5, 100 mM NaCl, 5% glycerol, 4% DMSO) with the ligand (40–80 µM, syringe:cell ratio 8:1) in the same buffer at 25 °C with a stirring rate of 75 rpm in 21-26 injections (1 × 1 µL, 20–25 × 3–4 µL). As control experiments, the test compounds were titrated to the buffer, and the buffer was titrated to the Nurr1 LBD protein under otherwise identical conditions. The vidofludimus-Nurr1 ITC data have been reported previously^23^. Ligand binding (vidofludimus, **1**, fluvastatin) to the Nurr1-I500W/M379W double mutant LBD was studied by titrating the recombinant protein (15 µM) in buffer (20 mM Tris pH 7.5, 100 mM NaCl, 5% glycerol, 3% DMSO) with the ligand (100 µM; syringe:cell ratio 6.67:1) in the same buffer at 25 °C with a stirring rate of 75 rpm in 16 injections (1 × 1 µL, 15 × 4 µL). As control experiments, the test compounds were titrated to the buffer, and the buffer was titrated to the Nurr1 LBD protein under otherwise identical conditions. Nurr1-RXR dimerization was studied by titrating RXRα LBD (15 µM) in buffer (50 mM Tris pH 7.4, 100 mM NaCl, 2% DMSO) with Nurr1 LBD (100 µM; syringe:cell ratio 6.67:1) in the same buffer at 25 °C with a stirring rate of 75 rpm in 21 injections (1 × 1 µL, 20×4 µL) in absence or presence of the ligands vidofludimus or **1**. Nurr1 homodimer dissociation was studied by titrating Nurr1 LBD (100 µM) in buffer (50 mM Tris pH 7.4, 100 mM NaCl, 2% DMSO) into the same buffer at 25 °C with a stirring rate of 75 rpm in 21 injections (1 × 1 µL, 20 × 4 µL) in absence or presence of the ligands vidofludimus or **1**. The ligands were added for the dimerization experiments at a fixed concentration (2-fold excess) to the titrator and titrant. The heats of the Nurr1-buffer titrations were analyzed with a dimer dissociation model. The Nurr1-RXR titrations were analyzed by subtracting the heats of the corresponding Nurr1-buffer titrations (i.e., Nurr1 homodimer dissociation) from the heats of the Nurr1-RXR titrations and analyzing the remaining heats with an independent binding model. It should be noted that this analysis of the heterodimer ITC studies data does not consider the contribution of RXR LBD homodimerization. As this effect can be expected to be consistent in all presented experiments, it should not affect the relative impact of the studied Nurr1 ligands on heterodimerization, while the absolute thermodynamic parameters (Supplementary Table 1) may reflect contributions of homodimer dissociation and heterodimer formation.

### Chemistry

#### General

All chemicals were of reagent grade, purchased from commercial sources (Enamine and BLDpharm), and used without further purification unless otherwise specified. All reactions were conducted in oven-dried Schlenk glassware under Ar atmosphere and in absolute solvents. Other solvents, especially for work-up procedures, were of reagent grade or purified by distillation (cyclohexane, EtOAc, EtOH). Reactions were monitored by thin layer chromatography (TLC) on TLC Silica gel 60 F_254_ aluminum sheets by Merck and visualized under ultraviolet light (254 nm) or by in-process LC/MS. Purification of compound **1** by preparative HPLC was performed on a puriFlash^®^ 5.250 system (Advion) using a uptisphere strategy C18-HQ prep-LC column (5 µM, 150 × 30 mm) and a gradient of H_2_O with 10–100% ACN (HPLC gradient grade). Mass spectra were obtained on a puriFlash^®^-CMS system (Advion) using atmospheric pressure chemical ionization (APCI). HRMS spectra were obtained with a Thermo Finnigan LTQ FT instrument using electron impact ionization (EI). NMR spectra were recorded on a Bruker Avance III HD 400 MHz spectrometer. Chemical shifts are reported in δ values (ppm), coupling constants (*J*) in hertz (Hz). The purity of compound **1** was determined by quantitative ^1^H NMR (qH NMR) according to the method described by Pauli et al.^48^ with internal calibration. To ensure accurate determination of peak area ratio, the qH NMR measurements were conducted under conditions allowing for complete relaxation. Ethyl 4-(dimethylamino)benzoate (LOT#BCCC6657, purity 99.63%) was used as internal standard in DMSO-*d*_6_.

*2-[(4-Bromo-2-fluorophenyl)carbamoyl]cyclopent-1-ene-1-carboxylic acid (****4****)*. 4-Bromo-2-fluoroaniline (**2**, 380 mg, 2.0 mmol, 1.0 eq) and 1-cyclopentene-1,2-dicarboxylic anhydride (**3**, 276 mg, 2.0 mmol, 1.0 eq) were dissolved in methylene chloride (10 mL) and stirred at ambient temperature for 16 h. Aqueous hydrochloric acid (10%, 20 mL) was added to the suspension, phases were separated, and the aqueous layer was extracted with methylene chloride (3x). The organic layers were combined, dried over Na_2_SO_4_, filtered, and evaporated to afford compound **4** (648 mg, 99%) as colorless solid. *R*_f_ (cyclohexane/EtOAc = 7:3 + 2% FA) = 0.38. MS (APCI-): *m*/*z* 325.6 ([M-H]^−^). ^1^H NMR (400 MHz, acetone-*d*_6_): δ = 10.61 (s, 1H), 8.19 (t, *J* = 8.5 Hz, 1H), 7.49–7.43 (m, 1H), 7.42–7.35 (m, 1H), 3.02–2.92 (m, 2H), 2.92–2.82 (m, 2H), 1.92 (p, *J* = 7.7 Hz, 2H) ppm. ^13^C NMR (101 MHz, acetone-*d*_6_): δ = 166.88, 164.60, 154.50 (d, *J* = 250.3 Hz), 146.88, 139.63, 128.42 (d, *J* = 3.7 Hz), 126.67 (d, *J* = 11.1 Hz), 125.62, 119.56 (d, *J* = 22.8 Hz), 117.19 (d, *J* = 8.7 Hz), 37.33, 37.03, 21.35 ppm.

*2-{[2’-(Benzyloxy)-3-fluoro-[1,1’-biphenyl]-4-yl]carbamoyl}cyclopent-1-ene-1-carboxylic acid (****1****)*. 2-[(4-Bromo-2-fluorophenyl)carbamoyl]cyclopent-1-ene-1-carboxylic acid (**4**, 66 mg, 0.20 mmol, 1.00 eq), 2-(benzyloxy)phenylboronic acid (**5**, 50 mg, 0.22 mmol, 1.20 eq) and Cs_2_CO_3_ (195 mg, 0.60 mmol, 3.00 eq) were placed in vacuum for 10 minutes. A solvent mixture of toluene (2 mL), EtOH (1.33 mL), and H_2_O (0.66 mL) was degassed by the freeze-pump-thaw method (3x) and added under Ar. Then XPhos-Pd-G2 (16 mg, 0.02 mmol, 0.10 eq) was added, and the reaction was stirred at 100 °C for 18 h. After cooling to rt, EtOAc (5 mL) and H_2_O (5 mL) were added, and the mixture was filtered through Celite. All solvents were removed under reduced pressure, and the resulting residue was suspended in aqueous HCl (10%, 10 mL). The aqueous mixture was extracted with EtOAc (3 × 10 mL). The organic layers were combined, dried over Na_2_SO_4_, filtered, and evaporated. The crude product was purified by HPLC to give compound **1** (56 mg, 65%) as a yellow solid. *R*_f_ (cyclohexane/EtOAc = 7:3 + 2% FA) = 0.63. MS (APCI + ): *m*/*z* 431.6 ([M + H]^+^). HRMS (EI + ): *m*/*z* calculated 431.1527 for [C_26_H_22_FNO_4_]^+•^, found: 431.1522 ([M]^+•^). ^1^H NMR (400 MHz, CD_2_Cl_2_) δ = 8.23 (t, *J* = 8.4 Hz, 1H), 8.13 (s, 1H), 7.48 (dd, *J* = 12.4, 1.9 Hz, 1H), 7.45–7.40 (m, 1H), 7.40–7.25 (m, 7H), 7.11–7.01 (m, 2H), 5.11 (s, 2H), 3.08–2.89 (m, 4H), 2.00 (p, *J* = 7.8 Hz, 2H) ppm. ^13^C NMR (101 MHz, CD_2_Cl_2_): δ = 165.12, 164.04, 155.92, 153.23 (d, *J* = 244.6 Hz), 148.89, 139.17, 138.05 (d, *J* = 8.1 Hz), 137.34, 131.00, 129.83, 129.21 (d, *J* = 1.9 Hz), 128.89, 128.24, 127.54, 126.20 (d, *J* = 3.1 Hz), 123.37 (d, *J* = 10.5 Hz), 122.66, 121.75, 116.80 (d, *J* = 20.0 Hz), 113.49, 70.83, 37.89, 36.16, 20.57 ppm, ^19^F{^1^H} NMR (376 MHz, CD_2_Cl_2_): δ = −130.20 ppm. qH NMR (400 MHz, DMSO-*d*_6_, Ethyl 4-(dimethylamino)benzoate as reference): purity = 99.1%. For NMR spectra of compound **1** see Supplementary Data 2.

### Computational procedures

#### Molecular docking

All calculations were performed with Schrödinger software version 2019-4. Chain E of the Nurr1 LBD (pdb id: 1ovl) was chosen and prepared for docking calculations using the Protein Preparation Wizard in Maestro. The center of the docking grid was set to −2.992, 1.941, −3.886. Docking was performed using GLIDE software^49,50^ in extra precision mode (XP). All parameters were left at the default values (XP; flexible ligands; sample ring conformations of ligand; add Epik^51^ state penalties to docking score; scaling of van der Waals radii = 0.8; post docking minimization of 5 poses per ligand; write out 5 poses per ligand). The ligands (vidofludimus and **1**) were converted to 3D using ligprep. The forcefield OPLS5 was used and the protonation between pH 6 to 8 was calculated with Epik.

#### Molecular dynamics

Molecular dynamics (MD) calculations were carried out in GROMACS Version 2024.4^52^. The OPLS-AA forcefield^53^ with TIP4P water model was used^54^, and the forcefield parameters for vidofludimus and **1** were generated by the LigParGen web server^55^. All simulated protein ligand complexes were put into a dodecahedral shaped unit cell, and six sodium ions were added resulting in a system without total charge. The solvated systems were equilibrated for 100 ps using NVT and NPT settings while restraining the coordinates of the protein and ligand. This resulted in system sizes for Nurr1-WT-vidofludimus: 12338 water molecules, cell sizes x = 8.3233, y = 8.3233, z = 5.88546; Nurr1-I500W-vidofludimus: 12248 water molecules, cell sizes x = 8.31028, y = 8.31028, z = 5.87625; WT-**1**: 15645 water molecules, cell sizes x = 8.95272, y = 8.95272, z = 6.33053; I500W-**1**: 14543 water molecules, cell sizes x = 8.74489, y = 8.74489, z = 6.18357. After equilibration, the MD simulation without any restraints was carried out for 200 ns. Each MD simulation was independently repeated four times. Starting temperatures and velocities for the atoms were set randomly by using gen_seed = -1 for the starting NVT simulations.

#### Sequence similarity search (SSS)

SSS was performed by NCBI blast+ Clustal Omega. For the Nurr1 DBD sequence as input, a score of 232.6 Bits was obtained for Nurr1 DBD (100% identity), 214.5 Bits for Nor1 DBD (94.2% identity), and 197.2 Bits for Nur77 DBD (86.1% identity). For the Nurr1 LBD sequence as input, the score was 475.7 Bits for Nurr1 LBD (100% identity), 301.2 Bits for Nor1 LBD (65.5% identity), and 329.3 Bits for Nur77 LBD (66.8% identity).

## Supplementary information

Supplementary Information

Description of Additional Supplementary Files

Supplementary Data 1

Supplementary Data 2

## Data Availability

All data supporting the results of this study are available from the corresponding author.
