# Supplementary material for: Structural and mechanistic profiling of Nurr1 modulation by vidofludimus enables structure-guided ligand design
Source: Commun Chem. 2025 May 21;8:159. doi: 10.1038/s42004-025-01553-8 (PMC12095788; doi:10.1038/s42004-025-01553-8)
Supplement: Supplementary file 1 — Supplementary Information [file 42004_2025_1553_MOESM1_ESM.pdf]

- Supplementary Information -

Structural and mechanistic profiling of Nurr1 modulation by vidofludimus enables structure-guided ligand design

Úrsula López-García[1], Jan Vietor[1], Julian A. Marschner[1], Jan Heering[2], Vasily Morozov[1], Thomas Wein[1], Daniel Merk[1]*

[1] Ludwig-Maximilians-Universität München, Department of Pharmacy, 81377 Munich, Germany

[2] Fraunhofer Institute for Translational Medicine and Pharmacology ITMP, 60596 Frankfurt, Germany

* daniel.merk@cup.lmu.de

Table of Contents

Supplementary Figures & Tables 2

Supplementary References 5

Supplemantary Figures & Tables

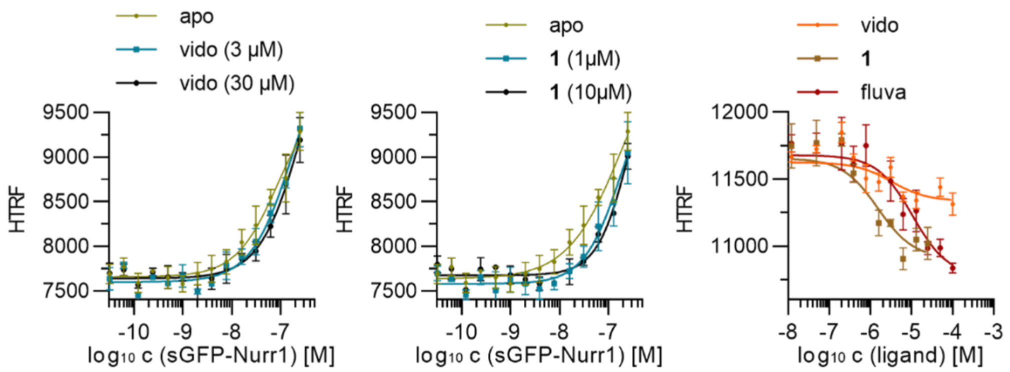

Supplementary Figure 1. Vidofludimus (vido), **1** and fluvastatin (fluva) slightly reduced recruitment of biotinylated CoREST peptide coupled to Tb-SA to the sGFP-labeled Nurr1 LBD in HTRF. Data are the mean±SD, N=3.

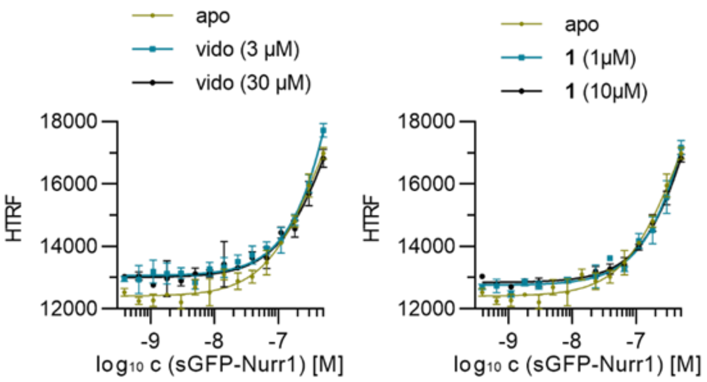

Supplementary Figure 2. Vidofludimus (vido) and **1** had no effect on the recruitment of biotinylated Lmx1b peptide coupled to Tb-SA recruitment to the sGFP-labeled Nurr1 LBD in HTRF. Data are the mean±SD, N=3.

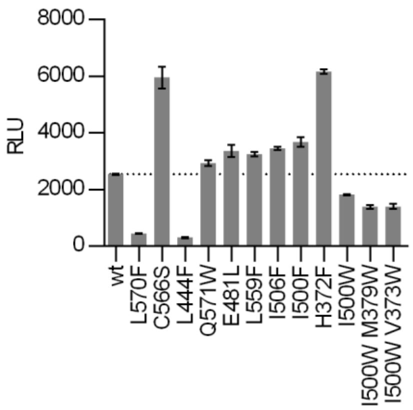

Supplementary Figure 3. Baseline activity of Nurr1 mutants compared to wild-type (wt) in Gal4-hybrid reporter gene assays. Data are the mean ± S.E.M.; n=3.

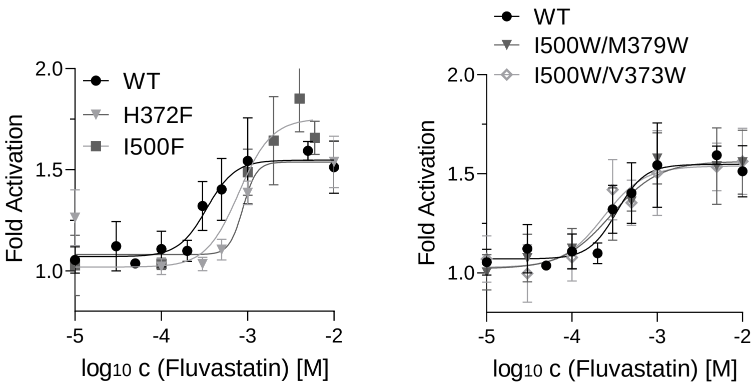

Supplementary Figure 4. Dose response curves of fluvastatin on wild-type Nurr1 and the mutants H372F, I500F, I500W/M379W, and I500W/V373W in Gal4-hybrid reporter gene assays. Data are the mean ± S.E.M.; n=3.

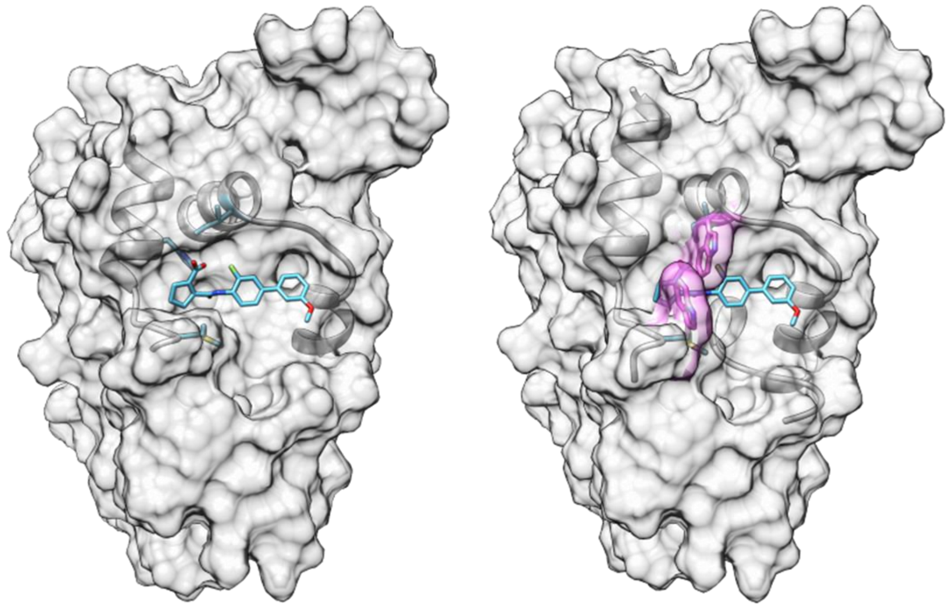

Supplementary Figure 5. Overview of the Nurr1 wt LBD structure (left) with vidofludimus (blue) shown in the proposed binding site. This proposed binding epitope is blocked in the Nurr1 I500W/M379W double mutant by the bulky tryptophane residues (magenta).

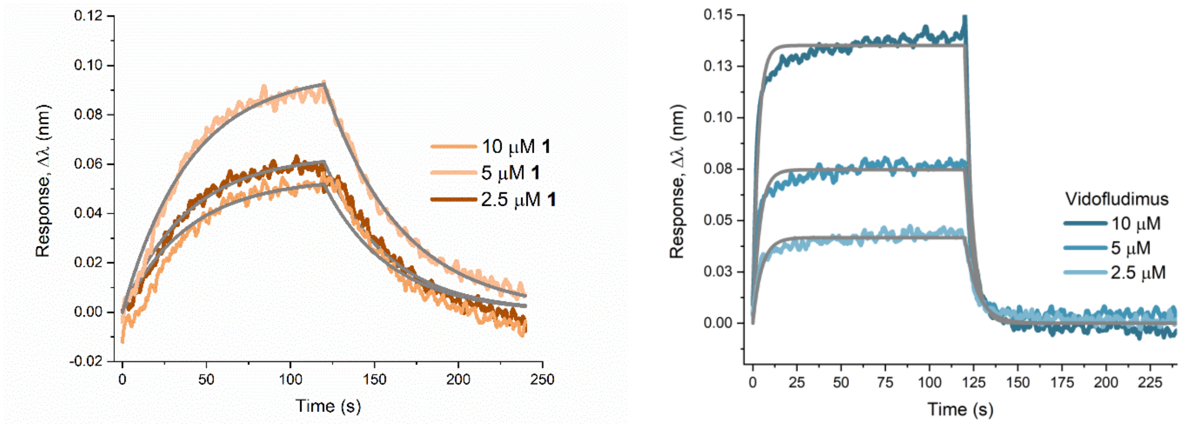

Supplementary Figure 6. Binding characteristics of **1** and vidofludimus to the Nurr1 LBD in biolayer interferometry (BLI). **1** displayed an improved dissociation rate (k_{off} 0.0224 s^{-1}, left) compared to vidofludimus (k_{off} 0.223 s^{-1}, right).

Supplementary Table 1. Experimental details and thermodynamic parameters of ITC experiments on dimerization of Nurr1 in absence and presence of vidofludimus (vido) or **1**. Data are the mean±SD, n=2.

experiment	syringe	cell	K_d(dimer) [µM]	n	ΔH [kcal/mol]	TΔS [kcal/mol]	c
Nurr1 LBD dimer dissociation (buffer)	Nurr1 LBD (100 µM)	buffer	7±1	-	-(28±4)	-(35±4)	-
Nurr1 LBD dimer dissociation (vido)	Nurr1 LBD (100 µM) + vido (200 µM)	buffer + vido (200 µM)	36±1	-	-(37±3)	-(43±3)	-
Nurr1 LBD dimer dissociation (**1**)	Nurr1 LBD (100 µM) + **1** (200 µM)	buffer + **1** (200 µM)	34±4	-	-(38±5)	-(44±5)	-
Nurr1 LBD – RXR LBD (buffer)	Nurr1 LBD (100 µM)	RXRα LBD (15 µM)	1.2±0.3	0.82±0.03	-(43±7)	-(35±7)	<10 [a]
Nurr1 LBD – RXR LBD (vido)	Nurr1 LBD (100 µM) + vido (200 µM)	RXRα LBD (15 µM) + vido (200 µM)	3.6±0.4	0.79±0.07	-(24±3)	-(17±3)	<10 [a]
Nurr1 LBD – RXR LBD (**1**)	Nurr1 LBD (100 µM) + **1** (200 µM)	RXRα LBD (15 µM) + **1** (200 µM)	2.9±0.2	0.98±0.09	-(21±1)	-(13±1)	<10 [a]

[a] Values for ΔH and TΔS should be interpreted with care for experiments with low value of c (see Suppl. Ref. [1]).

Supplementary Table 2. Experimental details and thermodynamic parameters of ITC experiments on binding of vidofludimus (vido), **1** and fluvastatin (fluva) to the Nurr1 wildtype and I500W/M379W LBD. Data are the mean±SD, n=2.

experiment	syringe	cell	Kd [µM]	n	ΔH [kcal/mol]	TΔS [kcal/mol]	c
vido-Nurr1 mutant LBD	vido (100 µM)	Nurr1 I500W/M379W LBD (15 µM)	(no binding)	-	-	-	-
**1**-Nurr1 LBD	**1** (50-100 µM)	Nurr1 LBD (5-15 µM)	0.11±0.03	1.00±0.04	-(15.0±1.9)	(-5.5±1.8)	>10/>100
**1**-Nurr1 mutant LBD	**1** (100 µM)	Nurr1 I500W/M379W LBD (15 µM)	(no binding)	-	-	-	-
fluva-Nurr1 LBD	fluva (100 µM)	Nurr1 LBD (15 µM)	0.6±0.2	0.90±0.04	-(4.8±0.4)	3.7±0.3	>10
fluva-Nurr1 mutant LBD	fluva (100 µM)	Nurr1 I500W/M379W LBD (15 µM)	0.49±0.02	1.09±0.04	-(2.6±0.4)	6.0±0.3	>10

Supplementary Table 3. Primer sequences used for Site-Directed Mutagenesis

Mutant	Forward Primer	Reverse Primer
L570F	5´ GCACACAGGGG**TT**CCAGCGCATT T-Pho 3´	5´ AAAGGGTACGAAGTTCTGGGAGCTTC-Pho 3´
C566S	5´ TCGTACCCTT**A**GCACACAGGG-Pho 3´	5´ AGTTCTGGGAGCTTCCCCAACAGTTT-Pho 3´
L444F	5´ GAATCAGCTTTCTT**T**GAACTGTTTGTC CT-Pho 3´	5´ AAAAAGCAGGTCTTGGTCGGC-Pho 3´
Q571F	5´ ACAGGGGCTA**TG**GCGCATTTTC-Pho 3´	5´ GTGCAAAGGGTACGAAGTTCTGG-Pho 3´
E481L	5´ CGTGGCTTTGGG**TT**ATGGATTGATTC-Pho 3´	5´ AACGCATTGCAACCTGTGCA-Pho 3´
L559F	5´ CTGTTGGGGAAG**T**TCCCAGAA CTTC-Pho 3´	5´ TTTGGACAAATAATTGGGGCGGTTC-Pho 3´
I506F	5´ CTTCTCCTGC**T**TTGCTGCCCT-Pho 3´	5´ GCAGAAATGTCGATGTTCATATTCTGCAAG-Pho 3´
I500F	5´ ATATGAACATCGAC**T**TTTCTGCCTTCTCC-Pho 3´	5´ TCTGCAAGTTGGAGGAGAATTCAACA-Pho 3´
H372F	5´ GTCAGGGCC**TT**TGTCGACTCCAACCCG 3´	5´ AGTCGACA**AA**GGCCCTGACGAGGGCAC 3´
I500W	5´ AACATCGAC**TGG**TCTGCCTTCTCCTGCATTGCTG3´	5´AGGCAGA**CCA**GTCGATGTTCATATTCTGCAAGTTGG 3´
V373W	5´ TCGTCAGGGCCCAT**TGG**GACTCCAACCCGGC3´	5´ GTTGGAGTC**CCA**ATGGGCCCTGACGAGGGCAC3´
M379W	5´ ACCCGGCT**TG**GACCAGCCTGGACTATTC3´	5´ GCTGGTC**CA**AGCCGGGTTGGAGTCGAC3´

Primers used for mutants L570F, C566S, L444F, Q571W, E481L, L559F, I559F, I506F and I500F are non-overlapping primers phosphorylated at the 3´end that contained the mutation in the forward primer. Primers used for mutants I500W, H372F, V373W and M379W were overlapping primers and both forward and reverse primers contained the desired mutation. Mutated residues are labeled in bold.

Supplementary Table 4. Fluorescein-labeled co-regulator peptides for HTRF assays

Co-regulator	Sequence	Source
nuclear receptor co-repressor (NCOR) ID1	Fluorescein-RTHRLITLADHICQIITQDFARN-OH	ThermoFisher Scientific
silencing mediator for retinoid and thyroid hormone receptor (SMRT) ID2	Fluorescein-HASTNMGLEAIIRKALMGKYDQW-OH	ThermoFisher Scientific
nuclear receptor co-activator 6 (NCoA6, also termed PRIPRAP250)	Fluorescein-VTLTSPLLVNLLQSDISAG-OH	ThermoFisher Scientific
nuclear receptor interacting protein 1 (NRIP1, also termed RIP140, interaction motif L6)	Fluorescein-SHQKVTLLQLLLGHKNEEN-OH	ThermoFisher Scientific
steroid receptor co-activator (SRC) 1-1	Fluorescein-KYSQTSHKLVQLLTTTAEQQL-OH	ThermoFisher Scientific
protein inhibitor of activated STAT protein gamma (PIASγ)	FITC-Ahx-MSFRVSDLQMLLGFVGRSK-OH	Suppl. Ref. [2]
REST corepressor 1 (CoREST)	Biotin-FDPAKLARRSQERDNLGMLV	Suppl, Ref. [3]
LIM homeobox transcription factor 1-beta (Lmx1b)	Biotin-QARVGNPIDRLYSMQSSYFAS	Suppl. Ref. [4]

Supplementary References

1. Turnbull, W. B. & Daranas, A. H. On the Value of c: Can Low Affinity Systems Be Studied by Isothermal Titration Calorimetry? *J. Am. Chem. Soc.* **125**, 14859–14866 (2003).

2. de Vera, I. M. S. *et al.* Identification of a Binding Site for Unsaturated Fatty Acids in the Orphan Nuclear Receptor Nurr1. *ACS Chem. Biol.* **11**, 1795–1799 (2016).

3. Saijo, K. *et al.* A Nurr1/CoREST Pathway in Microglia and Astrocytes Protects Dopaminergic Neurons from Inflammation-Induced Death. *Cell* **137**, 47–59 (2009).

4. Hoekstra, E. J., Mesman, S., de Munnik, W. A. & Smidt, M. P. LMX1B Is Part of a Transcriptional Complex with PSPC1 and PSF. *PLoS One* **8**, (2013).
