# Supplementary material for: Structural and mechanistic profiling of Nurr1 modulation by vidofludimus enables structure-guided ligand design
Source: Commun Chem. 2025 May 21;8:159. doi: 10.1038/s42004-025-01553-8 (PMC12095788; doi:10.1038/s42004-025-01553-8)
Supplement: Supplementary file 2 — Description of Additional Supplementary Files [file 42004_2025_1553_MOESM2_ESM.pdf]

Supplementary Data 1 contains coordinates of the MD simulations.

Supplementary Data 2 contains NMR spectra of compound **1**.
