# Supplementary material for: Structural and mechanistic profiling of Nurr1 modulation by vidofludimus enables structure-guided ligand design
Source: Commun Chem. 2025 May 21;8:159. doi: 10.1038/s42004-025-01553-8 (PMC12095788; doi:10.1038/s42004-025-01553-8)
Supplement: Supplementary file 4 — Supplementary Data 2 [file 42004_2025_1553_MOESM4_ESM.zip › Supplementary Data 2.pdf]

# - Supplementary Data – NMR Spectra

Structural and mechanistic profiling of Nurr1 modulation by vidofludimus enables structure-guided ligand design

Úrsula López-García[1], Jan Vietor[1], Julian A. Marschner[1], Jan Heering[2], Vasily Morozov[1], Thomas Wein[1], Daniel Merk[1]*

[1] Ludwig-Maximilians-Universität München, Department of Pharmacy, 81377 Munich, Germany

[2] Fraunhofer Institute for Translational Medicine and Pharmacology ITMP, 60596 Frankfurt, Germany

* daniel.merk@cup.lmu.de

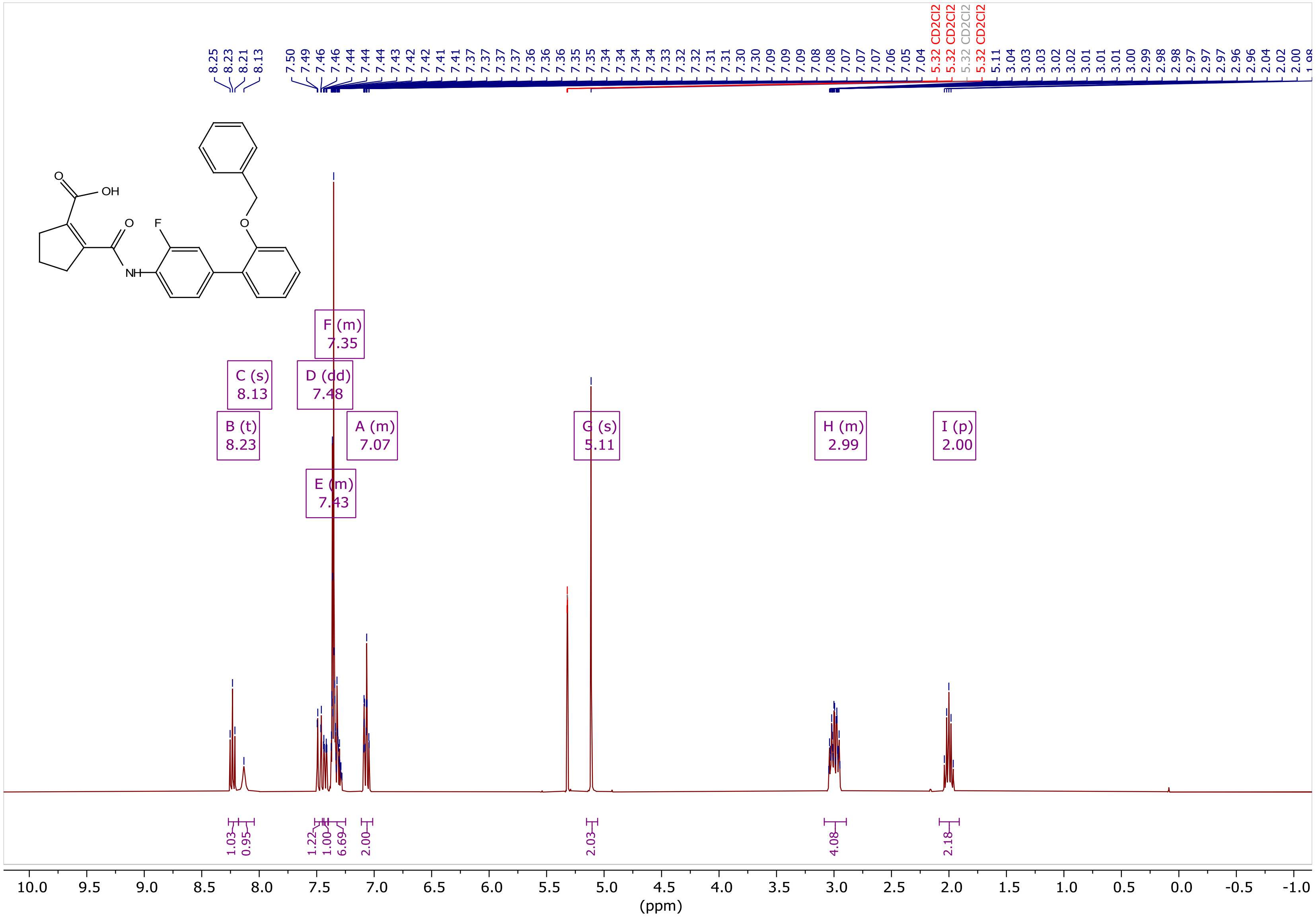

^{1}H NMR spectrum (400 MHz, CD_2Cl_2) of compound **1**

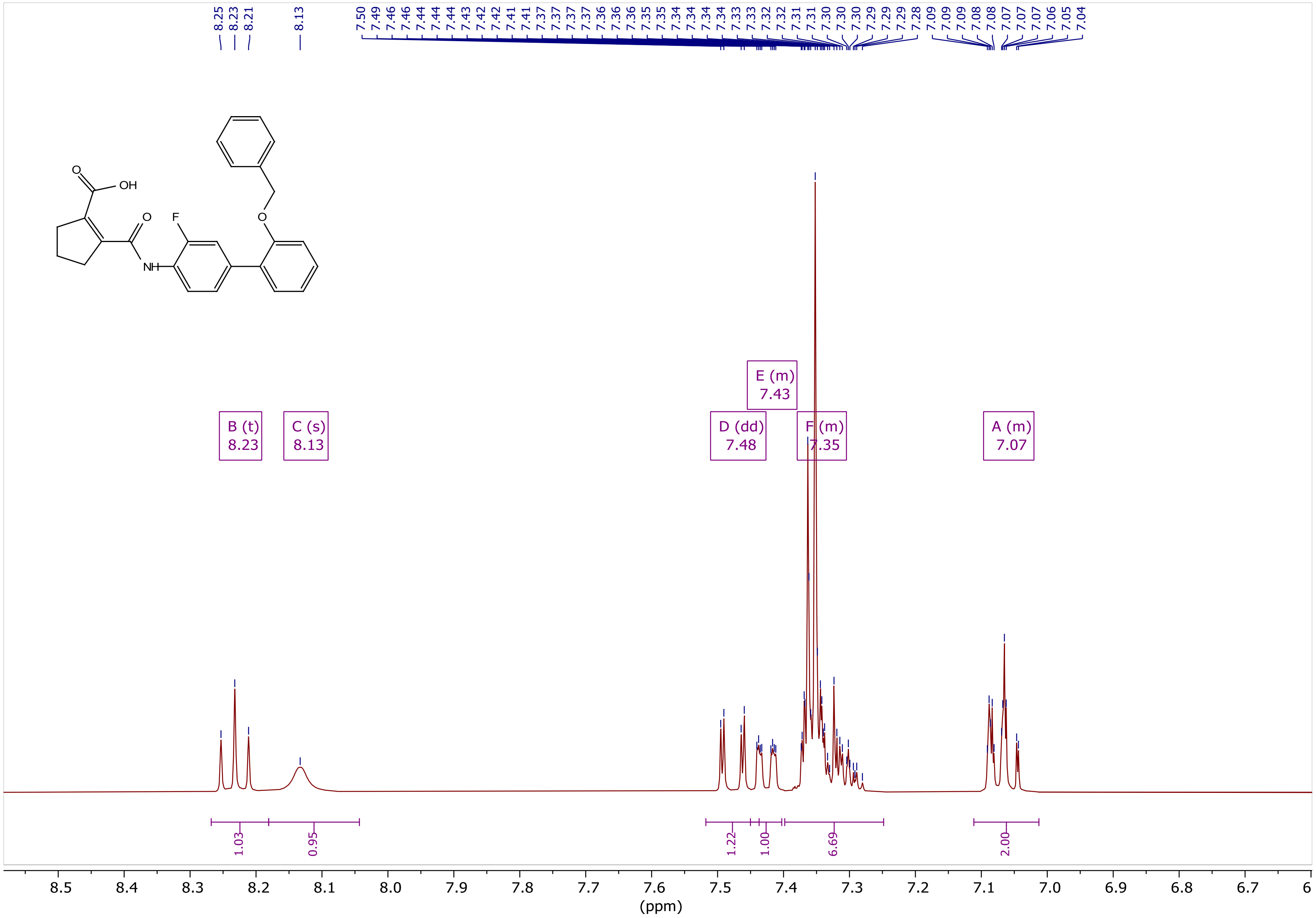

Enlarged section of the ^1H NMR spectrum (400 MHz, CD_2Cl_2) of compound **1**

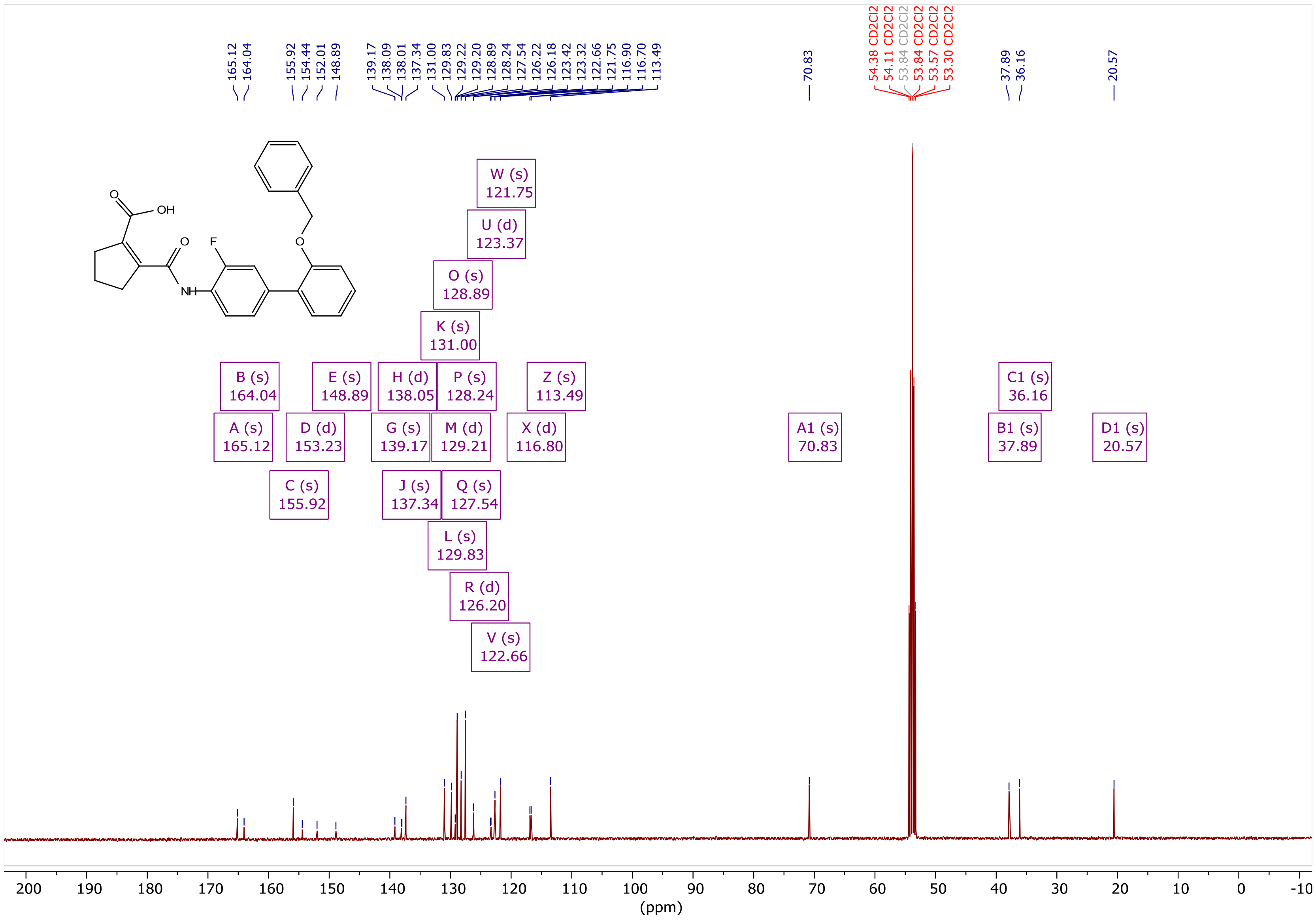

$^{13}C\{^{1}H\}$ NMR spectrum (101 MHz, CD_2Cl_2) of compound **1**

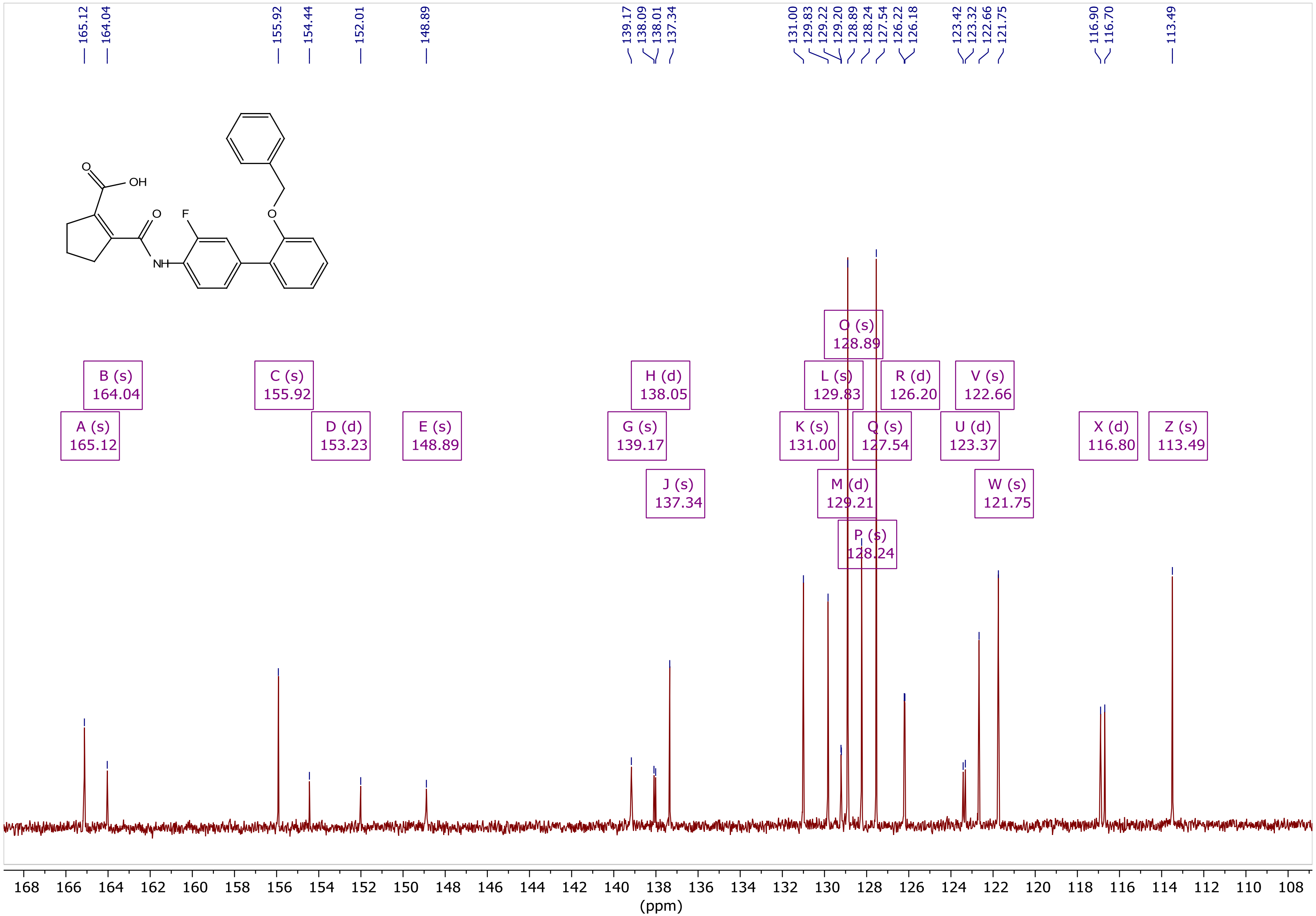

Enlarged section of the $^{13}C\{^{1}H\}$ NMR spectrum (101 MHz, CD_2Cl_2) of compound **1**

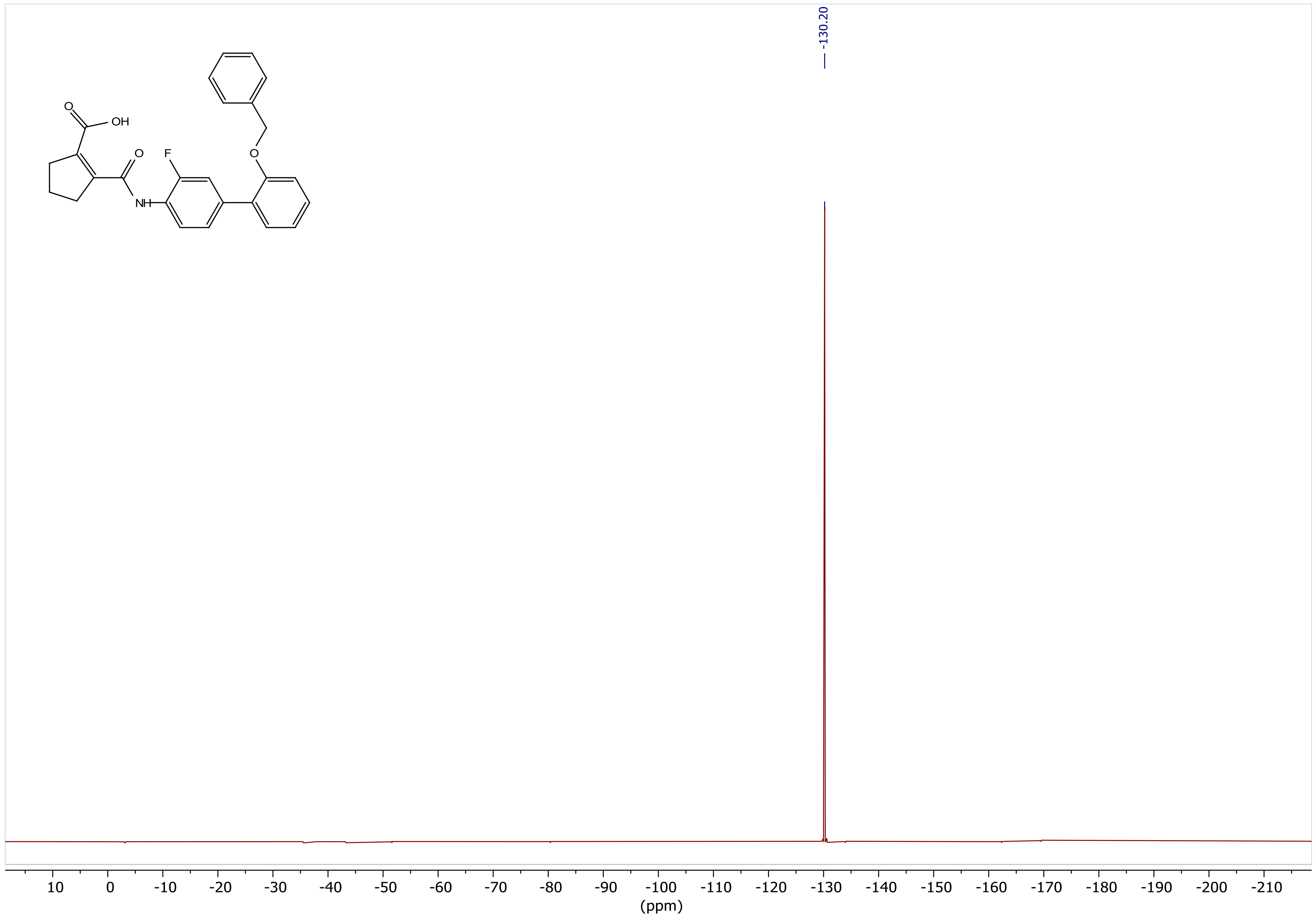

$^{19}F\{^1H\}$ NMR spectrum (376 MHz, CD_2Cl_2) of compound **1**

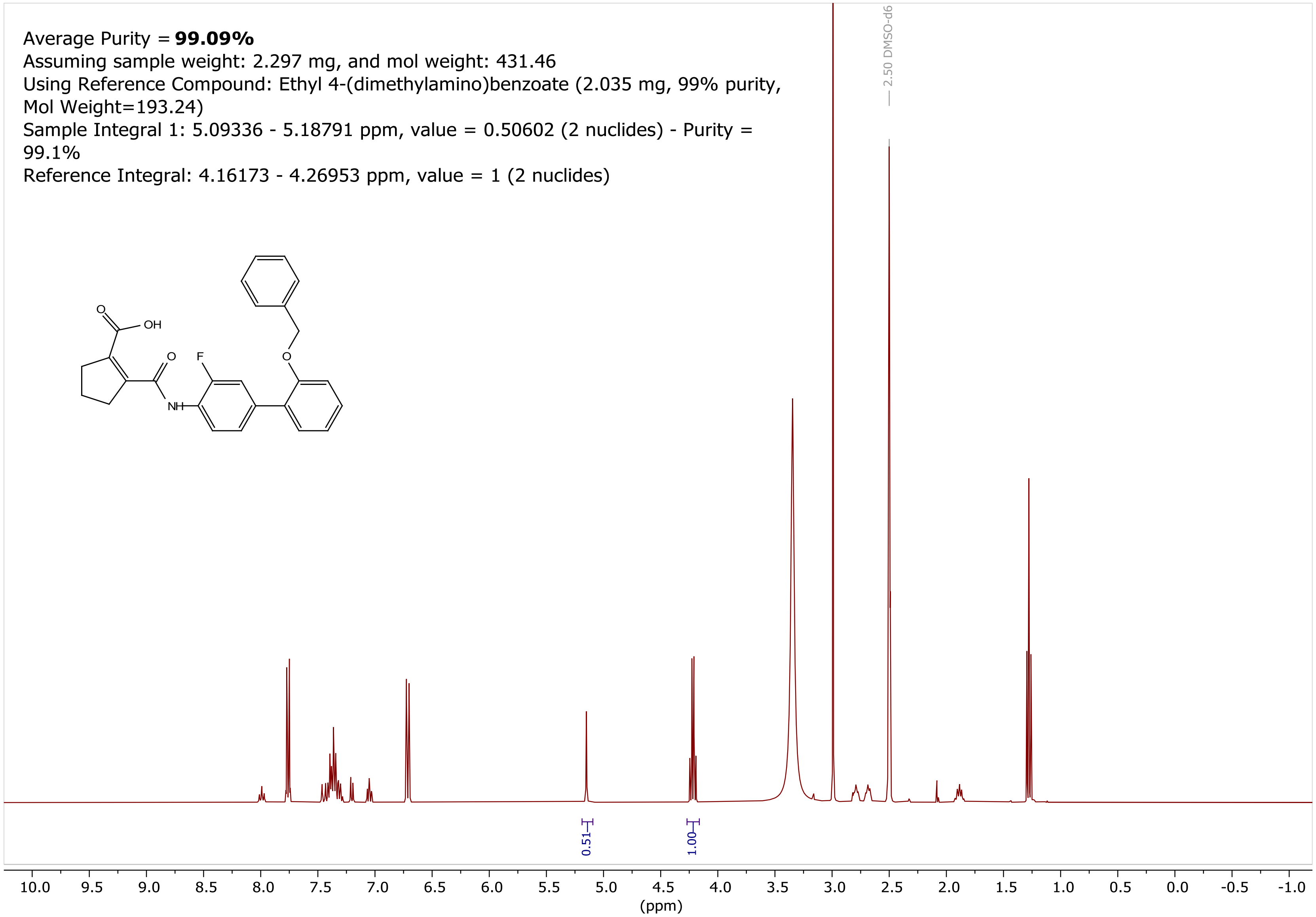

qH NMR spectrum (400 MHz, DMSO-d_6) of compound **1**
